# Complementary Strategies of Hydraulic Variability and Conservative Stomatal Regulation Enable Widespread Distributions in a Heterogeneous Karst Landscape

**DOI:** 10.1002/ece3.72744

**Published:** 2025-12-17

**Authors:** Xiaojuan Wang, Xuefeng Ma, Rilian Ma, Bin Wang, Fengsen Tan, Qiwei Zhang

**Affiliations:** ^1^ Key Laboratory of Ecology of Rare and Endangered Species and Environmental Protection (Ministry of Education) & Guangxi Key Laboratory of Landscape Resources Conservation and Sustainable Utilization in Lijiang River Basin Guangxi Normal University Guilin Guangxi China; ^2^ University Engineering Research Center of Bioinformation and Genetic Improvement of Specialty Crops in Guangxi Guilin Guangxi China; ^3^ College of Life Sciences Guangxi Normal University Guilin Guangxi China; ^4^ Guangxi Institute of Botany Chinese Academy of Science Guilin Guangxi China; ^5^ Engineering Research Center of Masson Pine of Guangxi & Guangxi key Laboratory of Superior Timber Trees Resource Cultivation Guangxi Forestry Research Institute Nanning China

**Keywords:** hydraulic safety, hydraulic traits, karst forest, stomatal regulation, variability

## Abstract

Karst ecosystems display pronounced elevational heterogeneity driven by steep water and soil gradients, fostering niche differentiation as a biodiversity maintenance mechanism. While hydraulic trait divergence underpins such distribution patterns, certain species thrive across diverse microhabitats. Intraspecific variability in drought‐resistant hydraulic traits may enable such broad‐niche species to exploit habitat heterogeneity, but whether it is a key mechanism in karst ecosystems is unclear. Specifically, locally widespread species lacking hydraulic variability may compensate through conservative stomatal regulation to maintain hydraulic safety, thereby colonizing extreme arid hilltops. For three widespread woody species in a tropical karst forest (*Delavaya toxocarpa*, *Tirpitzia ovoidea*, *Litsea glutinosa*), we quantified the intraspecific variability of key hydraulic traits (leaf area, LA; leaf mass per area, LMA; leaf turgor loss point, Ψ_TLP_; stem vulnerability to embolism, Ψ_12_ and Ψ_50_; hydraulic conductivity, *K*_s_ and diameter of stem vessels, *D*_h_) and the stringency of stomatal regulation (isohydricity index, *σ*; hydroscape area) along a water availability gradient. Results revealed that as water availability decreased, all three species shifted toward more conservative stomatal regulation (decreasing *σ* and hydroscapes area), but their hydraulic trait adjustment patterns differed. *D. toxocarpa* and *T. ovoidea* exhibited high intraspecific variability in leaf (reduced LA, increased LMA, more negative Ψ_TLP_) and stem traits (declining *D*_h_, *K*_s_, Ψ_12_, Ψ_50_), enabling colonization of xeric hilltops via hydraulic safety prioritization. Their high variability indices (*PPI*_TLP_, *PPI*_Ψ50_) were consistent with superior community importance. Conversely, *L. glutinosa* maintained static hydraulic traits and relied primarily on a consistently conservative stomatal strategy (maintaining a stable hydraulic safety margin, HSM_50_), which facilitates wide distribution but limits competitiveness in karst forest, as reflected in its low importance value (a composite metric of abundance, basal area, and distribution). These findings demonstrate that variability‐mediated tradeoffs between hydraulic efficiency and safety, coupled with leaf‐stem trait coordination, are key drivers of niche partitioning in karst forests. The study advances understanding of plant adaptive strategies in stochastic environments, emphasizing the role of trait variability in ecological dominance under resource heterogeneity.

## Graphical abstract

Steep water‐soil gradients in karst create niche differentiation, yet how widespread trees span microhabitats remains unclear. Along a moisture gradient, Delavaya toxocarpa and Tirpitzia ovoidea colonize xeric hilltops through high hydraulic trait variability that prioritizes hydraulic safety, whereas Litsea glutinosa depends on conservative stomatal control with static traits, achieving broad but low‐competitive distribution. Trait variability and coordinated leaf‐stem tradeoffs drive niche partitioning in karst forests, representing a key adaptive mechanism under resource heterogeneity.

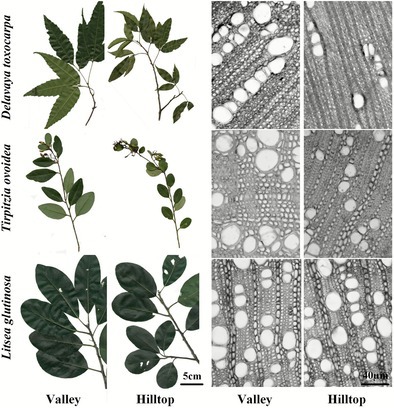

## 1 Introduction

Water availability is a critical determinant of species distribution in terrestrial ecosystems (Choat et al. 2007; Pfautsch et al. 2016). This limitation arises because reduced water uptake by roots elevates xylem sap tension, thereby increasing the risk of air entry into vascular conduits (i.e., embolism) (Tyree and Zimmermann 2002). To survive in water deficit, woody plants have evolved multiple characteristics to preserve the integrity of the hydraulic system, including stomatal regulation to reduce transpirational water loss, structural modifications of xylem architecture to enhance embolism resistance, and leaf shedding to reduce overall water demand (Delzon 2015; Pivovaroff et al. 2016).

The karst landform in southwest China was identified as one of the world's largest continuous limestone zones (Yuan 1992). This region was characterized by Peak‐Cluster Depression, where clustered limestone hills surround central depressions that funnel surface runoff (Wang et al. 2016). This unique geomorphology drives a special hydrological process. Most of the rain as concentrated flow infiltrates underground through rocky conduits and fractures, while residual precipitation forms surface runoff that transports colluvial deposits into valley bottoms (Williams 2008). Consequently, soil depth and hydrological connectivity decrease from valley to hilltop, paralleled by declining soil moisture (Williams 2008; Hartmann et al. 2014). Such extreme heterogeneity generates differential drought stress, exposing hilltop trees to a higher risk of hydraulic failure compared to those in depression valleys (Geekiyanage et al. 2019; Zhang et al. 2021). The pronounced moisture gradient across tropical karst landscapes promotes most plant distribution clustering (Zhang et al. 2012). This spatial stratification exhibits strong elevational dependence; for example, *Boniodendron minius* predominantly occupies xeric hilltops and upper slopes, *Ficus hispida* is restricted to valley and lower shapes (Wang et al. 2016; Guo et al. 2016). This niche differentiation reflects interspecific divergence in hydraulic strategies among co‐occurring species (Zhang et al. 2021). To persist in water‐limited hilltop microhabitats, evergreen species typically rely on a suite of traits and mechanisms that confer resistance to hydraulic dysfunction. These include traits such as more negative Ψ_50_ (the water potential inducing 50% loss of maximum hydraulic transport function in leaf or branch), more negative Ψ_TLP_ (the water potential at turgor loss point), wider *HSM* (hydraulic safety margin), and smaller vessels, as well as protective mechanisms like hydraulic segmentation to limit the spread of embolism (Choat et al. 2007; Blackman et al. 2012; Pfautsch et al. 2016; Zhang et al. 2021).

Despite contrasting water availability, certain woody species in tropical karst forest exhibit broad distributions spanning humid valleys to xeric hilltops (Wang et al. 2016). Confronting this ecological challenge, the observed intraspecific variability across microhabitats represents a potential adaptive strategy to navigate such environmental heterogeneity. While numerous studies have established significant correlations between plant hydraulic traits (including Ψ_50_, Ψ_TLP_, *HSM*, *D*_h_, and vulnerability segmentation) and habitat‐specific water availability (Pockman and Sperry 2000; Baltzer et al. 2008; Bartlett et al. 2012; Zhang et al. 2021), intraspecific variability in some of these traits remains contentious (Beikircher and Mayr 2009; Cardoso et al. 2018; Lobo et al. 2018; Fuchs et al. 2021), yet how they enable adaptation to karst environments is poorly understood. Furthermore, the relevance of other key drought‐coping traits must be considered within the specific constraints of karst ecosystems. For instance, although deep rooting can decouple embolism resistance from habitat aridity (Choat et al. 2018), this strategy is likely limited in the shallow, rocky soils of karst hilltops, where roots are primarily confined to topsoil layers and rock surfaces (Eggemeyer and Schwinning 2009; Elkington et al. 2014). Similarly, woody species on xeric karst outcrops are usually small in stature (Wang et al. 2016; Dammeyer et al. 2016), and the limited water storage capacity in their stem tissues may buffer diurnal water deficits but is insufficient to overcome prolonged drought (Phillips et al. 2003; Meinzer et al. 2009; Pratt and Jacobsen 2017). Consequently, within the constrained morphological and physiological context of karst hilltops, demonstrated adaptations such as hydraulic segmentation (Zhang et al. 2021; Huang et al. 2024), along with the stomatal regulation observed in our study, are critical for surviving extreme aridity. This underscores that when core hydraulic traits exhibit limited variability, widespread karst species depend heavily on such compensatory mechanisms to maintain hydraulic safety (Delzon 2015). Thus, survival in these heterogeneous environments is best understood as the integrated outcome of complex adaptive strategies, where the synergy among traits ultimately defines a species' ecological niche (Reich 2014; Martínez‐Vilalta et al. 2019).

Specifically, stomatal regulation varies along a continuum of drought‐response strategies, ranging from more isohydric to more anisohydric behavior (Tardieu and Simonneau 1998; Martínez‐Vilalta et al. 2014; Hochberg et al. 2018). The more conservative isohydric behavior contributes to maintaining a larger hydraulic safety margin (*HSM*) (Skelton et al. 2015; Pivovaroff et al. 2018), a critical adaptation for xeric hilltop survival (Zhang et al. 2021; Huang et al. 2024).

*Delavaya toxocarpa*, *Tirpitzia ovoidea*, and *Litsea glutinosa* are evergreen species widely distributed across heterogeneous microhabitats in the karst landform. While interspecific trait variation is known to facilitate niche partitioning at the community level (Zhang et al. 2021), the intraspecific mechanisms allowing particular species to thrive across steep environmental gradients are poorly understood. To test the hypothesis that widespread species compensate for limited variability in core hydraulic traits through conservative stomatal regulation, we quantified their correlated hydraulic traits (*D*_h_, Ψ_12_, Ψ_50_, Ψ_TLP_, *K*_S_, HSM) and stomatal regulation (*σ* parameter) along moisture gradients. This study identifies the adaptive strategy of widespread karst species, providing physiological criteria for selecting drought‐resilient species in karst rocky desertification restoration.

## 2 Methods and Materials

### 2.1 Study Site and Woody Species

The research was conducted in Pingxiang City, Guangxi Zhuang Autonomous Region, southern China (106°44′44″ E, 22°07′22″ N). This region features classic karst topography, with elevation ranging from 246 to 444 m and slope gradients ranging from gentle valleys (3°) to steep hillsides (65°). Based on comprehensive topographical and edaphic characteristics, the “peak‐depression” could be divided into three distinct microhabitats: (1) Valleys occur at elevations below 300 m and are characterized by minimal bedrock exposure of less than 10%, deep soils approximately 70 cm thick, and gentle slopes ranging from 3° to 20°. (2) Slopes are found at elevations between 300 and 380 m and feature intermediate bedrock exposure of approximately 60%, moderate soil depth up to 30 cm, and steep gradients ranging from 20° to 65°. (3) Hilltops are located at elevations above 380 m and are distinguished by extensive bedrock exposure exceeding 95%, shallow soils less than 10 cm deep, and a complex topography featuring predominantly very steep slopes interspersed with occasional narrow, relatively flat ridges. In the dry season, volumetric soil water content measured at 5–10 cm depth was significantly lower at hilltops compared to valleys and slopes, while no significant difference was detected between the latter two microhabitats. However, the potential water storage capacity decreased from slopes to hilltops due to reductions in soil depth and continuity (Figure S1). Climatically, this region is under the influence of the Pacific monsoon, with a mean annual temperature and precipitation of 22°C and 1470 mm, respectively. There is a distinct dry season from October to May, with 30% annual rainfall occurring during this period.

The three selected species were evergreen, with a relatively wide elevational distribution. For each of the three species (*D. toxocarpa*, *T. ovoidea* , and *L. glutinosa* ), five mature, healthy individuals per microhabitat (valley, slope, hilltop) were selected for measurements of anatomy, pressure–volume curves, and stomatal regulation. Due to the higher branch demand for constructing vulnerability curves, additional individuals were sampled specifically for this purpose, bringing the total to *c*. 10 individuals per species per microhabitat for hydraulic measurements. Overall, the study incorporated 45 individuals for core trait analysis, with supplemental sampling for hydraulic vulnerability assessment. From each species, sun‐exposed branches from the upper canopy were sampled for various measurements. The basic characteristics of the sampled individuals, including their taxonomic family, DBH, height, and the sample sizes (*n*) for each specific measurement, are detailed in Table S1. All measurements were carried out during the dry season.

### 2.2 Leaf Trait Measurements

Leaf area (LA) was measured for 50 leaves per species in every niche using ImageJ software (v 1.50) after being scanned. Leaves were then oven‐dried at 72°C for 72 h. Leaf mass per area (LMA, g/m^2^) was calculated as dry mass divided by LA.

The leaf turgor loss point (Ψ_TLP_) was determined via pressure–volume (P‐V) curves generated through the bench drying method (Tyree and Hammel 1972). The resulting P‐V curves are provided in Figure S2. Sampling was performed before dawn (05:00–07:00). Five sun‐exposed terminal branches (*c*. 50 cm) were collected from five individuals per species across three microhabitats (valley, slope, hilltop). Following excision, branch bases were recut underwater and transported to the laboratory in water‐filled containers, with leaf‐bearing tips wrapped in light‐impermeable bags. Before measurements, all samples were rehydrated for 2 h. During controlled dehydration, fresh mass and water potential (Ψ_leaf_) were measured periodically using an analytical balance (±0.0001 g) and pressure chamber (PMS Model 1505D), respectively. Concurrently, LA and dry mass were quantified. Pressure–volume (P‐V) curves were constructed by plotting Ψ against relative water content (RWC).

### 2.3 Stem Trait Measurements

To determine Ψ_50s_, maximum vessel length (MVL) was assessed via the air infiltration technique first. Three intact branches (> 2 m) per species and microhabitat were harvested. Each branch was submerged and pressurized with 60 kPa air at the distal end. Sequential 1 cm proximal cuts were made underwater until air bubbles emerged. MVL was recorded as the remaining segment length plus 0.5 cm (data shown in Table S1). For vulnerability curve construction, a separate set of branches was used. For each curve, multiple branches of twice the MVL length were collected at predawn. Each branch was used for a single dehydration time point measurement (Tyree and Sperry 1989; Wheeler et al. 2013). Following excision, branches were sealed in light‐impermeable bags with moist towels and transported to the laboratory immediately. After re‐cutting underwater, the harvested branches were rehydrated for 2 h. Subsequently, the cut ends were wrapped in wax and parafilm (PM996; BEMIS). Then, all branches were subjected to a dehydration process on laboratory benches. Several leaves on branches were encapsulated with aluminum foil to inhibit evaporative water loss. After allowing the samples to air‐dry on the bench for varying durations (ranging from 0.5–20 h depending on the target water potential), the branches were placed in light‐impermeable bags containing moist towels for approximately 1 h to equilibrate. The stem water potential (Ψ_stem_) was determined by averaging the measurements from two wrapped leaves. In cases where the Ψ_stem_ values of these leaves differed by less than 0.20 MPa, the branches were used for subsequent experiments; otherwise, they were re‐equilibrated. For hydraulic conductivity measurements, each dehydrated branch (representing one time point) was processed individually. Subsequently, to avoid cutting artifacts, the corresponding branches were submerged in water and cut a segment at the end (Wheeler et al. 2013). Two hours later, stem segments exceeding the MVL by 20% were cut off underwater and attached to an apparatus to quantify the mass flow rate (J_V_, kg/s) and axial pressure gradient (Δ*P*/Δ*L*, MPa/m). An ultra‐filtered (particle size < 0.2 μm), degassed solution of KCl with a concentration of 20 mmol·L^−1^ was employed as the perfusion fluid. The initial hydraulic conductivity (*K*_i_) was calculated as:

$$K_{i}=J_{V}/\left(\Delta P/\Delta L\right)$$

Then, to remove air embolism, the segment was flushed with the KCl solution at 0.15 MPa pressure for 20 min. Finally, the maximum stem hydraulic conductivity (*K*_max_) was determined by using the above‐mentioned apparatus. The percentage loss of conductivity (PLC) was calculated as:

$$\mathrm{PLC}=100\;\left(K_{\max}-K_{i}\right)/K_{\max}$$

A series of Ψ_stem_ measurements and their corresponding PLC value were obtained using the bench dehydration. The vulnerability curves were parametrized through sigmoidal regression analyses.

Meanwhile, we randomly selected five branches per species and microhabitat to determine the sapwood specific hydraulic conductivity (*K*_s_, kg·m^−1^·s^−1^·MPa^−1^). This was calculated by dividing *K*_max_ by the functional sapwood cross‐sectional area. To ensure this, the bark was removed at both ends of the segment before measurement. The functional sapwood area was determined at the segment's midpoint by subtracting the area of the nonconductive pith (heartwood) from the total stem cross‐sectional area.

For anatomical analysis, transverse sections were prepared using a precision sliding microtome (SM2010R; Leica). Sections were obtained from the middle portion of the stem segments previously used for hydraulic conductivity measurements (five independent replicates per species × microhabitat combination). To control for the known gradient in vessel size with distance from the branch tip, all sampled segments were of a similar diameter (typically 1.5 cm). Sequential histochemical staining was conducted using 0.5% safranin O solution (Sangon Biotech, Shanghai, China) for 10 min, followed by 1% alcian blue 8GX (Sangon Biotech) for 3 min. Digital imaging was performed using a light microscope (DM2500; Leica) equipped with a camera (MC190 HD; Leica). To determine the hydraulically weighted vessel diameter (*D*_h_, μm), four fields per section were acquired using a 40 × objective lens. *D*_h_ was calculated according to the formula:

$$D_{h}={\left(\sum D^{4}/N\right)}^{1/4}$$

where *D* represented the equivalent circular diameter of vessels; *N* represented the number of vessels measured (Tyree and Zimmermann 2002).

### 2.4 Stomatal Regulation Strategy

To quantify the stringency of stomatal regulation, we employed two complementary approaches. First, we calculated the isohydric behavior index (*σ*) as the slope of the linear regression between predawn (Ψ_PD_) and midday (Ψ_MD_) leaf water potentials (Martínez‐Vilalta et al. 2014). The *σ* value theoretically defines a continuum of stomatal behavior: *σ* = 0 indicates strict isohydric behavior; 0 < *σ* < 1 indicates partial isohydric behavior; *σ* = 1 indicates strict anisohydric behavior; and *σ* > 1 indicates extreme anisohydric behavior. Second, we characterized the “hydroscape” for each species following Meinzer et al. (2016). The hydroscape is defined as the area in the Ψ_MD_‐Ψ_PD_ plane bounded by the regression line of Ψ_MD_ vs. Ψ_PD_, the *y*‐axis, and the 1:1 line. This area represents the landscape of water potential over which stomata effectively regulate plant water status, with a larger hydroscape area indicating a broader operational range and a more anisohydric strategy (Figure S4).

Throughout rainless periods in the dry season, predawn (Ψ_PD_) and midday (Ψ_MD_) leaf water potentials were longitudinally monitored. The study employed five replicate individuals per species within each of the three microhabitats. For each individual at each time point, measurements were conducted on two leaves, serving as technical replicates. The monitoring was carried out at intervals of 1–2 days over an approximately 20‐day period. Ψ_PD_ measurements were conducted between 05:00 and 06:00 h using the above‐mentioned pressure chamber, followed by Ψ_MD_ determinations from 12:30 to 14:30 h, with duplicate measurements per individual to ensure data robustness. Moreover, to determine species‐specific hydraulic safety margins (*HSM*), five mature, healthy individuals of each species were selected at the terminal phase of the dry season. Two sun‐exposed leaves per individual were encapsulated in aluminum foil sleeves 12 h before sampling to avoid transpiration. The minimum water potential (Ψ_min_) was measured on the foil‐protected leaves excised between 12:30 and 14:30 h. The hydraulic safety margin was calculated at two critical thresholds following the convention of Meinzer et al. (2009): *HSM*_12_ = Ψ_min_−Ψ_12_, representing the safety margin at the onset of hydraulic failure, and *HSM*_50_ = Ψ_min_−Ψ_50_, representing the margin before severe hydraulic dysfunction occurs.

### 2.5 Statistical Analysis

All statistical and regression analyses were performed in R version 3.5.3 (www.r‐project.org). Vulnerability curves were fitted using the *fitplc* package, from which Ψ_12_ and Ψ_50_ were calculated. Kernel density distribution plots of vessel diameters were generated using the *ggplot2* package to visualize size‐class variations. The variability indices of key hydraulic parameters, including Ψ_TLP_, *K*_s_, Ψ_50_, and *HSM*, were calculated following Valladares et al. (2000). Specifically, for each species and parameter, we first calculated the mean value for each of the three microhabitats (*n* = 5 individuals per microhabitat). The variability index (*PPI*) for each parameter was derived as the difference between the highest of these three mean values and the lowest of these three mean values across three microhabitats, divided by the highest of these three mean values:

$$\mathrm{PPI}=\left({\text{Mean}}_{\max}-{\text{Mean}}_{\min}\right)/{\text{Mean}}_{\max}$$

## 3 Results

### 3.1 Leaf Trait Responses to the Microhabitat Moisture Gradient

**FIGURE 1:**
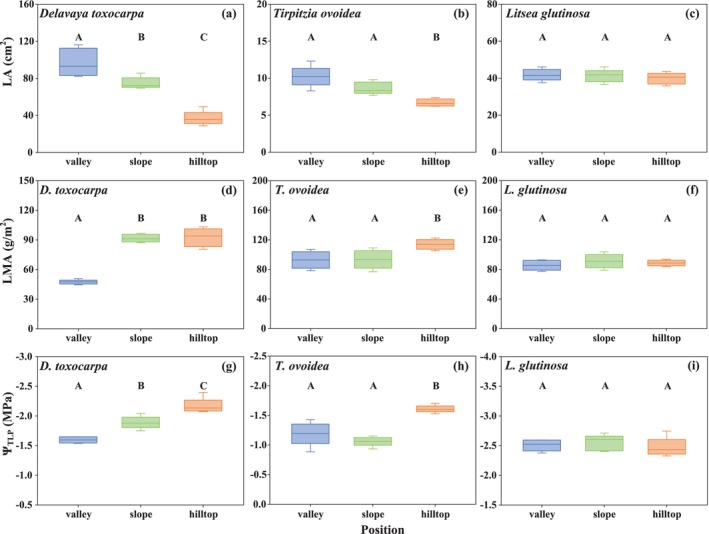
Leaf trait responses to the moisture gradient in (a, d, g) *Delavaya toxocarpa*, (b, e, h) *Tirpitzia ovoidea*, and (c, f, i) *Litsea glutinosa*. Traits shown are leaf area (LA), leaf mass per area (LMA), and leaf water potential at turgor loss point (Ψ_TLP_). Different letters indicate statistically significant differences among microhabitats (*p* < 0.05).

Despite variations in edaphic water availability across microhabitats (Figure S1), three woody species exhibited divergent leaf trait responses to the moisture gradient. *D. toxocarpa* demonstrated the most responsive leaf economics. From the moist valley to the dry hilltop, LA decreased substantially (*p* < 0.05; Figure 1a; Figure 5; Table S2), while leaf mass per area (LMA) increased significantly (*p* < 0.05; Figure 1d; Table S2). Concurrently, its Ψ_TLP_ became significantly more negative from the valley to the hilltop (*p* < 0.05; Figure 1g; Table S2). *T. ovoidea* displayed moderate variability. Its LA showed a significant decrease only at the driest hilltop site (*p* < 0.05; Figure 1b; Table S2), while LMA increased significantly at the driest hilltop site compared to lower positions (*p* < 0.01; Figure 1e; Table S2). The Ψ_TLP_ remained stable between the valley and slope but declined significantly at the hilltop (*p* < 0.05; Figure 1e; Table S2). In stark contrast, *L. glutinosa* exhibited highly conservative leaf traits, with no significant variations in LA, LMA, or Ψ_TLP_ across the three microhabitats (*p* > 0.05; Figure 1c,f,I; Figure 5; Table S2). For leaf drought tolerance (Ψ_TLP_), *D. toxocarpa* and *T. ovoidea* exhibited considerable variability (*PPI*_TLP_ = 0.26 and 0.34, respectively), whereas *L. glutinosa* showed minimal plasticity (*PPI*_TLP_ = 0.02) (Table S2).

### 3.2 Coordinated Adjustments in Stem Hydraulic Architecture

**FIGURE 2:**
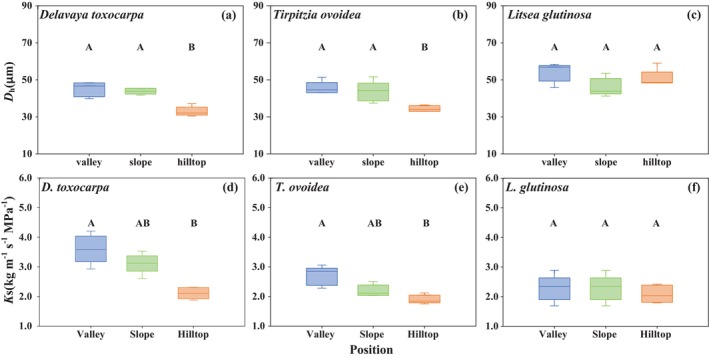
Variation patterns in hydraulic vessel diameter (*D*_h_) and specific hydraulic conductivity of stem xylem (*K*s) of (a, d) *D. toxocarpa*; (b, e) *T. ovoidea* ; and (c, f) *L. glutinosa* . Different letters indicate significant differences.

**FIGURE 3:**
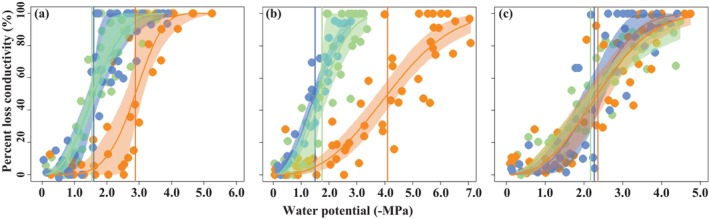
Stem vulnerability curves of (a) *D. toxocarpa*; (b) *T. ovoidea* ; and (c) *L. glutinosa* . Orange, green, and blue indicate hilltop, slope, and foot, respectively. The vertical solid lines indicate the water potential inducing a 50% loss of the maximum stem hydraulic conductivity. The shaded area indicates the 95% confidence interval.

The variation patterns in stem hydraulic traits closely paralleled those observed in foliar traits. The more variable species, *D. toxocarpa* and *T. ovoidea* , showed significant shifts in hydraulic architecture at the dry hilltop. Both species exhibited significant reductions in *K*_s_ (*p* < 0.05; Figure 2d,e; Table S2). For these species, this functional shift was associated with a significant reduction in *D*_h_ (*p* < 0.05; Figure 2a,b; Figure 5; Table S2). Concurrently, they showed a marked increase in xylem embolism resistance (i.e., more negative Ψ₅₀ values) (*p* < 0.05; Figure 3a,b; Table S2). Conversely, *L. glutinosa* maintained stable stem hydraulic traits across all microhabitats, showing no significant differences in *K*_s_, Ψ_50_, or *D*_h_ (*p* > 0.05; Figure 2c,f; Figure 3c; Figure 5; Table S2). Stem hydraulic traits revealed a divergent pattern of intraspecific variability among the species. *D. toxocarpa* exhibited the highest variability in hydraulic conductivity (*PPI*_Ks_ = 0.41), whereas *L. glutinosa* showed minimal variation (*PPI*_Ks_ = 0.14). In contrast, *T. ovoidea* displayed the most extreme variability in embolism resistance (*PPI*_Ψ50_ = 0.65) (Table S2), highlighting its distinct strategy of drastically reinforcing xylem safety under drought conditions.

### 3.3 Shifts in the Stringency of Stomatal Regulation

**FIGURE 4:**
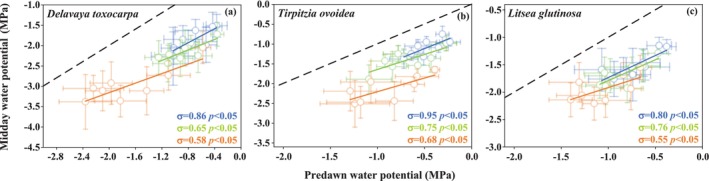
Stomatal regulation along moisture gradients of (a) *D. toxocarpa*; (b) *T. ovoidea* ; and (c) *L. glutinosa* . *σ*, the isohydric behavior index. Orange, green, and blue indicate hilltop, slope, and foot, respectively.

All three species showed a significant decrease in *σ* from the moist valley to the dry hilltop (Figure 4; Table S2), indicating a shift toward more stringent stomatal regulation in drier microhabitats. Notably, all *σ* values fell within the range of 0 to 1, indicating that all three species employ a strategy of partial isohydric regulation across the environmental gradient rather than operating at the theoretical extremes of the spectrum.

**FIGURE 5:**
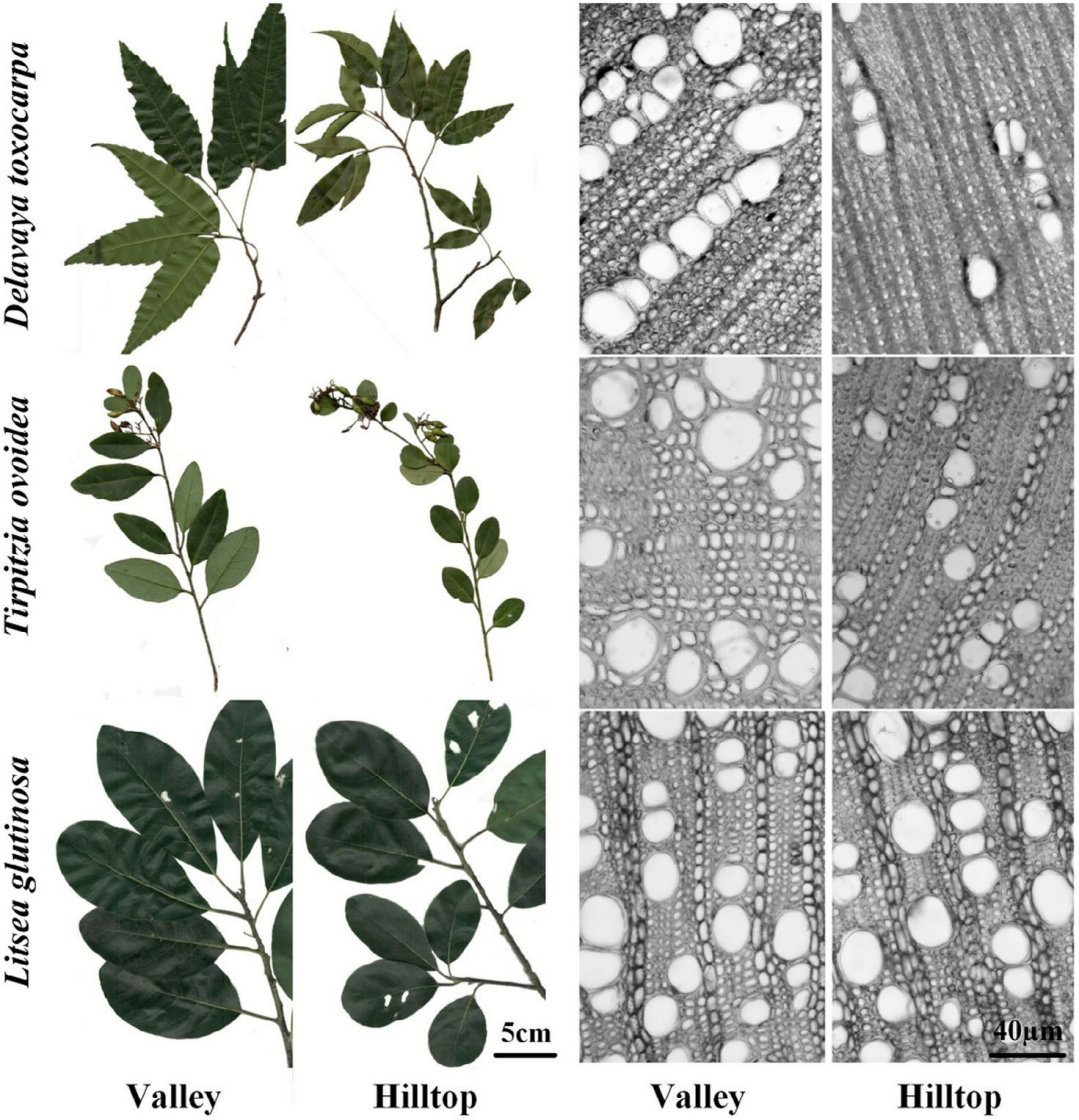
Representative morphology and anatomy of the three study species across microhabitats.

The hydroscape became progressively smaller from the valley to the hilltop in *D. toxocarpa* (5.37, 3.55, 4.66 MPa^2^) and *T. ovoidea* (4.52, 1.62, 3.68 MPa^2^). In contrast, the inherently conservative *L. glutinosa* maintained a consistently small hydroscape across all sites (2.19, 2.21, 2.07 MPa^2^) (Table S2). These coordinated adjustments in the stringency of stomatal regulation and xylem hydraulics resulted in distinct hydraulic safety margin (HSM) patterns. For HSM_12_, values were narrow and often negative across all three species and microhabitats. In contrast, HSM_50_ increased at the hilltop in *D. toxocarpa* (0.27 MPa) and *T. ovoidea* (2.02 MPa), while *L. glutinosa* maintained positive HSM_50_ values (ranging from 0.56 to 0.91 MPa) across all positions with minimal variation (Table S2).

## 4 Discussion

### 4.1 The Variability of Hydraulic Traits Is Jointly Regulated by Phylogenetic Constraints and Ecological Context

In karst ecosystems, despite a narrow altitudinal range of approximately 200 m across three microhabitats, pronounced heterogeneity in water availability was observed. To adapt to this water availability gradient, the three species exhibited divergent strategies. *D. toxocarpa* and *T. ovoidea* occupying xeric hilltops demonstrated variability‐driven tradeoffs, prioritizing hydraulic safety (wider *HSM* and lower Ψ_50_) at the expense of conductivity efficiency (reduced *D*_h_ and *K*_s_). In contrast, *L. glutinosa* maintained conservative hydraulic traits across microhabitats, showing minimal adjustments in Ψ_50_ or *K*_s_. These findings align with the interspecific variability in hydraulic trait conservatism reported in previous studies. For instance, Beikircher and Mayr (2009) observed significant plasticity in Ψ_50_ in *Ligustrum vulgare* and *Viburnum lantana* under moisture gradients, whereas Lobo et al. (2018) and Fuchs et al. (2021) documented limited trait adjustments in other taxa. This discrepancy underscores that hydraulic conservatism is phylogenetically constrained and ecologically context‐dependent, reflecting species‐specific evolutionary adaptations.

Furthermore, *L. glutinosa* is not only distributed in karst forests but also widely occurs in nonkarst regions with higher water availability (compared to karst areas) (Yu et al. 2017). However, whether its observed low variability in hydraulic traits represents an inherent phylogenetic constraint or a localized adaptive response to karst‐specific stressors remains unresolved. To validate this, comparative studies incorporating nonkarst populations are essential. Such investigations would clarify whether the conservative hydraulic strategy of *L. glutinosa* is a fixed trait across its distribution or a context‐dependent adaptation shaped by karst aridity.

It is important to note that the observed intraspecific variability in hydraulic traits across microhabitats could result from a combination of phenotypic plasticity and potential genetic differentiation among populations, although the latter was not assessed in this study.

### 4.2 The Variability of Hydraulic Architecture Coordinates With Stomatal Regulation Strategies to Safeguard Hydraulic Safety Under Arid Hilltop Conditions

As soil water availability decreased, all three species progressively shifted toward more conservative stomatal regulation, which was consistent with observations by Domec and Johnson (2012) and Hochberg et al. (2018). This shift was quantitatively reflected in a narrowing of the hydroscape from the moist valley to the dry slope. However, at the most arid hilltop, the hydroscape of the more variable species (*D. toxocarpa* and *T. ovoidea* ) showed a slight re‐widening. This nonlinear pattern suggests that after an initial tightening of regulation to enhance hydraulic safety, these species may partially relax stomatal constraints to maximize carbon assimilation when xylem embolism resistance is sufficiently high, representing a dynamic balance between carbon gain and hydraulic safety (Hochberg et al. 2018; Anderegg et al. 2018).

By tightening stomatal control in concert with shifts toward more embolism‐resistant xylem, plants effectively increase their *HSM*_50_. This enables them to operate farther from the threshold of embolism formation, thereby reducing susceptibility to hydraulic failure under extreme drought (Meinzer et al. 2009; Pivovaroff et al. 2018). Previous studies have demonstrated that robust drought resistance is essential for karst plants to adapt to arid hilltop environments (Zhang et al. 2021). By incorporating stomatal water regulation measurements, this study highlights the critical role of *HSM*_*50*_ in ensuring survival under such extreme conditions (Skelton et al. 2015).

Arid hilltop environments impose three compounding constraints: intensified drought stress from shallow soils and rapid water loss (Williams 2008; Zhang et al. 2021), high damage and even die‐off of species employing riskier stomatal strategies during droughts, regeneration bottlenecks driven by low seed germination success, primarily caused by exposed bedrock and limited soil accumulation (Zhang et al. 2012; Guo et al. 2016). These pressures collectively drive the evolutionary prioritization of conservative strategies, even in phenotypically rigid species like *L. glutinosa* . By maintaining positive *HSM*_50_ values, such species balance embolism avoidance with slow but sustainable growth, as exemplified by *L. glutinosa's* lowest Ψ_TLP_, a trait that aligns with the ‘slow’ strategy of the leaf economics spectrum characterized by conservative resource investment and delayed returns (Wright et al. 2004; Zhu et al. 2018). This equilibrium reflects natural selection in resource‐limited karst systems, where hydraulic safety outweighs ephemeral growth efficiency, as catastrophic embolism risks preclude opportunistic resource exploitation. However, the protection afforded by this strategy may be insufficient against the most extreme conditions.

A focus on the onset of embolism (Ψ_12_) reveals a more complex scenario. Our analysis shows that *HSM*_12_ was consistently narrow (< 1 MPa) and frequently negative across microhabitats. Notably, at the slope position, even the *HSM*_50_ of *D. toxocarpa* fell into negative territory (−0.26 MPa), indicating that its operational water potentials transiently crossed the theoretical threshold for a 50% loss of hydraulic conductivity. The survival of plants despite this recurrent exposure to irreversible damage poses a physiological paradox that is resolved by the hypothesis of hydraulic segmentation (Tyree and Ewers 1991; Zhang et al. 2021; Huang et al. 2024). This mechanism, which involves the sacrificial embolism of peripheral organs to isolate damage, finds some support in a previous study (Zhang et al. 2021), as well as the observed trend of more frequent re‐equilibration issues in hilltop populations. As a crucial adaptation in karst plants (Zhang et al. 2021; Huang et al. 2024), hydraulic segmentation is therefore an essential mechanism, acting as an ultimate “fuse” to manage the risk indicated by negative *HSM*. In conclusion, achieving hydraulic safety in the heterogeneous karst environment is not reliant on a single strategy but on an integrated, hierarchical approach.

### 4.3 Hydraulic Trait Variability Reflects Trade‐Offs Between Efficiency and Safety

The variability of hydraulic architecture in *D. toxocarpa* and *T. ovoidea* was driven by contrasting adjustments in vessel diameter, which directly shaped their *K*_s_. *D*. toxocarpa exhibited a progressive decline in vessel diameter along the slope gradient, with slope populations developing distinctive dimorphic vessels (Figure S3). However, the persistence of larger vessels, despite their heightened vulnerability to embolism, limited hydraulic safety (Tyree and Zimmermann 2002; Sperry et al. 2008). This is because embolism in a few large conduits disproportionately reduced whole‐stem conductivity (Sperry et al. 2008). This explains the lack of significant separation in vulnerability curves between foot and slope populations. In contrast, *T. ovoidea* maintained stable vessel diameters across foot and slope habitats (Figure S3), likely due to sufficient hydraulic safety margins (*HSM*_50_ > 0), and only exhibited variability under extreme hilltop drought. A similar pattern has been observed in *Pinus canariensis* , where plasticity in Ψ_50_ occurs exclusively at the driest end of the aridity gradient (López et al. 2016). These species‐specific patterns were consistent with the broader framework of efficiency‐safety trade‐offs (Gleason et al. 2015). While *D. toxocarpa* prioritized gradual acclimation to moderate drought, *T. ovoidea* conserved structural integrity until critical thresholds, reflecting divergent evolutionary solutions to karst heterogeneity.

### 4.4 Leaf Trait Coordination Reinforces Stem Hydraulic Variability

Adjustments in stem hydraulic traits were paralleled by coordinated shifts in leaf morphology and physiology. In *D. toxocarpa* and *T. ovoidea* , increased LMA and more negative Ψ_TLP_ collectively signify a transition from “fast” to “slow” resource‐use strategies (Wright et al. 2004; Zhu et al. 2018). This shift prioritizes drought resistance over photosynthetic capacity (Bartlett et al. 2012; Fortunel et al. 2018). Such a trade‐off reflects the physiological constraints imposed by the coupling of hydraulic efficiency and photosynthetic rates (Brodribb and Feild 2000; Brodribb et al. 2003). Moreover, plant hydraulic efficiency critically regulates leaf transpiration rates (Meinzer et al. 1999; Tyree and Zimmermann 2002; Manzoni et al. 2013), which serve as a primary mechanism for mitigating thermal and radiative stress during the daytime, particularly under water‐limited conditions (Gates 1965). Larger leaves exhibit thicker boundary layers, reducing convective heat exchange with ambient air while increasing radiative energy absorption (Gates 1965; Leigh et al. 2017). Consequently, higher transpirational demand in larger leaves creates a functional conflict when hydraulic efficiency declines. This explains the coordinated reduction in leaf area observed in *D. toxocarpa* and *T. ovoidea* across aridity gradients.

### 4.5 Variability in Drought‐Resistant Hydraulic Traits Underlies Community Competitiveness of Karst Plants

Previous studies demonstrate that functional trait plasticity governs plant colonization capacity in novel environments (Matesanz et al. 2012) and enhances competitive performance (Feng et al. 2011), ultimately facilitating ecological niche expansion under fluctuating environmental conditions (Valladares et al. 2000). In water‐limited karst ecosystems (Guo et al. 2016; Geekiyanage et al. 2019), hydraulic adjustments that maximize soil water uptake while maintaining hydraulic safety are critical for surviving in heterogeneous habitats. Field observations from the nearby Nonggang 15‐ha karst forest dynamics plot (Wang et al. 2016) revealed a hierarchical pattern in community importance values among the three species. Under equivalent altitude and hydrothermal regimes, *D. toxocarpa* exhibited the highest importance (rank 59), followed by *T. ovoidea* (rank 76) and *L. glutinosa* (rank 159). This rank order strongly correlated with drought tolerance traits' intraspecific variability (*PPI*_TLP_ and *PPI*_Ψ50_), highlighting the adaptive significance of hydraulic flexibility in karst plant communities. Structural variability enables a single species to achieve strategy diversification, fostering niche diversification. For instance, *T. ovoidea* 's ability to modulate vessel diameter (ΔD_h_ = 11.19 μm) allows it to occupy both mesic slope and xeric hilltop, effectively expanding its ecological amplitude. Such variability‐driven niche partitioning underscores why hydraulic adaptability, rather than static trait values, predicts success in stochastic environments (Valladares et al. 2000).

Our findings that *D. toxocarpa* and *T. ovoidea* exhibited high variability in key hydraulic traits align with the concept of “niche breadth” via phenotypic integration. Their ability to adjust both leaf and stem economics (e.g., increased LMA, more negative Ψ_TLP_, and reduced *K*_s_) allows them to function effectively across a range of water availabilities. This intraspecific variability in drought‐related traits likely represents a key mechanism enabling these habitat generalists to achieve a wide ecological niche in heterogeneous karst landscapes (Valladares et al. 2007). In contrast, the static strategy of *L. glutinosa* , while successful in stable environments, may constrain its niche to more mesic microsites, as reflected in its lower community importance value. This underscores that niche partitioning in stochastic environments is governed not by static trait values alone, but by the ability to dynamically adjust and coordinate multiple traits in response to environmental cues.

## 5 Conclusion

This study demonstrates that plants employ divergent strategies to colonize heterogeneous karst habitats. *D. toxocarpa* and *T. ovoidea* relied on high hydraulic trait variability and dynamic stomatal regulation to enhance drought resistance, whereas *L. glutinosa* succeeded through static traits and consistently conservative stomatal control. These findings highlight that niche partitioning in karst forests is shaped not only by fixed trait differences but also by species‐specific capacities for physiological adjustment. Our results provide a functional basis for selecting resilient species in karst restoration, emphasizing the role of hydraulic and stomatal flexibility in adapting to environmental heterogeneity.

## Author Contributions

**Xiaojuan Wang:** conceptualization (equal), data curation (equal), formal analysis (equal), investigation (lead), software (equal), validation (equal), writing – original draft (lead). **Xuefeng Ma:** investigation (equal), project administration (equal), software (supporting), visualization (equal). **Rilian Ma:** data curation (equal), formal analysis (supporting), investigation (supporting), methodology (equal), software (equal). **Bin Wang:** data curation (equal), investigation (equal). **Fengsen Tan:** conceptualization (equal), data curation (equal), investigation (equal), software (lead), validation (lead), writing – review and editing (lead). **Qiwei Zhang:** data curation (equal), funding acquisition (lead), methodology (equal), resources (equal), software (equal), supervision (lead), validation (equal), writing – original draft (equal).

## Funding

This work was supported by the Guangxi Young Elite Scientist Sponsorship Program, GXYESS2025221 and National Natural Science Foundation of China, 32060330, 32260265.

## Conflicts of Interest

The authors declare no conflicts of interest.

## Supporting information


**Figure S1:** Edaphic characteristics across the three microhabitats. (a) Volumetric soil water content measured at 5–10 cm depth during the dry season; (b) Percentage of bedrock exposure visually estimated within each microhabitat; (c) Soil depth measured from the surface to the refusal layer. Different lowercase letters above the boxes indicate significant differences among microhabitats (*p* < 0.05).

**Figure S2:** Pressure–volume curves for (a, b, c) *Delavaya toxocarpa*, (d, e, f) *Tirpitzia ovoidea*, and (g, h, i) *Litsea glutinosa*. Microhabitats are distinguished by color: valley (blue), slope (green), hilltop (yellow). The dashed vertical line denotes the leaf water potential at turgor loss point.

**Figure S3:** Frequency distribution of vessel diameters of (a) *D. toxocarpa*; (b) *T. ovoidea* ; and (c) *L. glutinosa* . Orange, green, and blue indicate hilltop, slope, and foot, respectively.

**Figure S4:** Hydroscape area for each species across the three microhabitats. Panels (a, b, c) represent *Delavaya toxocarpa*, (d, e, f) *Tirpitzia ovoidea*, and (g, h, i) *Litsea glutinosa*. Microhabitats are distinguished by color: valley (blue), slope (green), hilltop (yellow).

**Data S1:** Methods.

**Table S1:** Basic characteristics of sampled trees, sample size, and measured maximum vessel length across microhabitats.

**Table S2:** Summary of hydraulic traits and variability indices for three woody species across microhabitat gradients.

## Data Availability

The data used in this paper are available in the supplementary files and on the Open Science Framework (DOI: https://osf.io/mesw9/overview?view_only=ceae54ebe2e242eb92cd390443c86d8c).
