# Supplementary figures and images for: Complementary Strategies of Hydraulic Variability and Conservative Stomatal Regulation Enable Widespread Distributions in a Heterogeneous Karst Landscape
Source: Ecol Evol. 2025 Dec 17;15(12):e72744. doi: 10.1002/ece3.72744 (PMC12711600; doi:10.1002/ece3.72744)

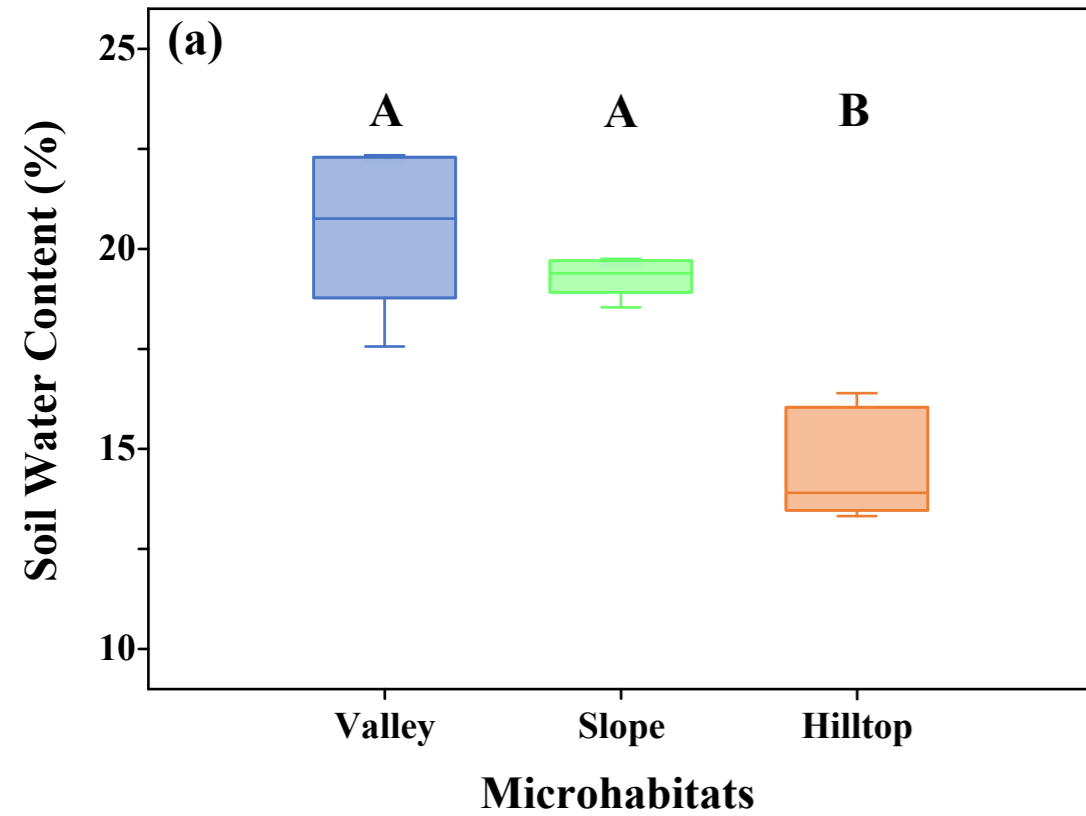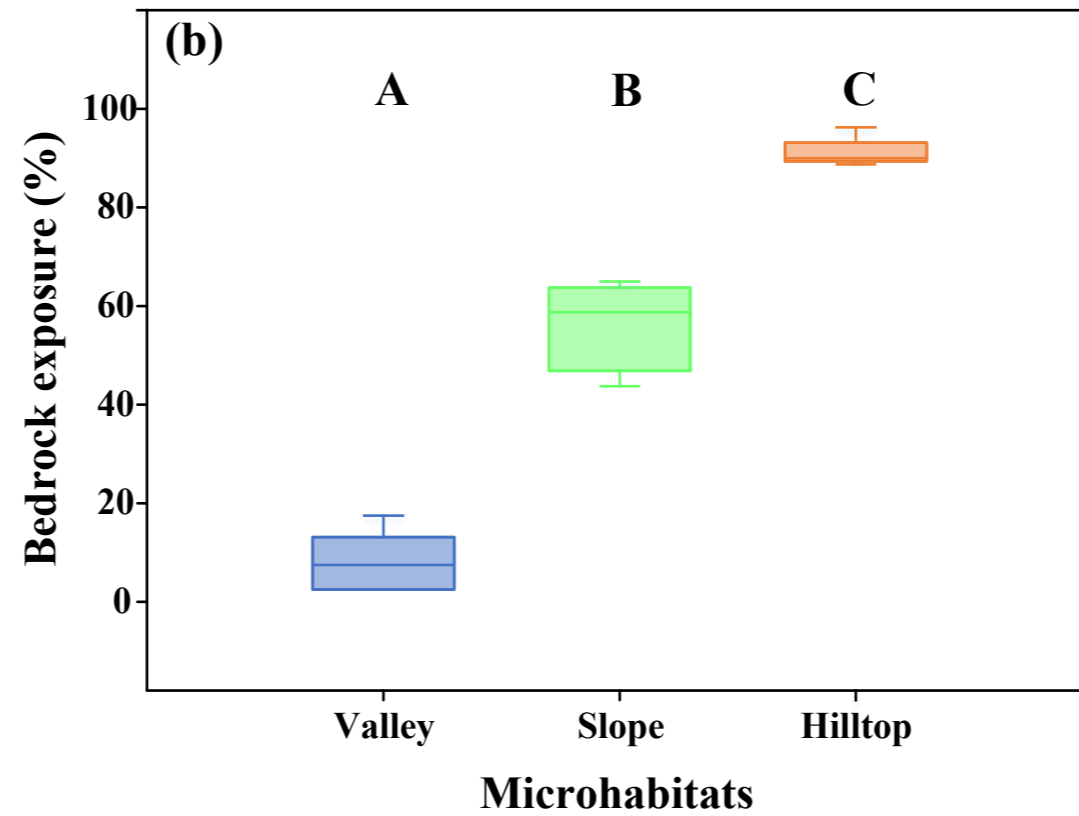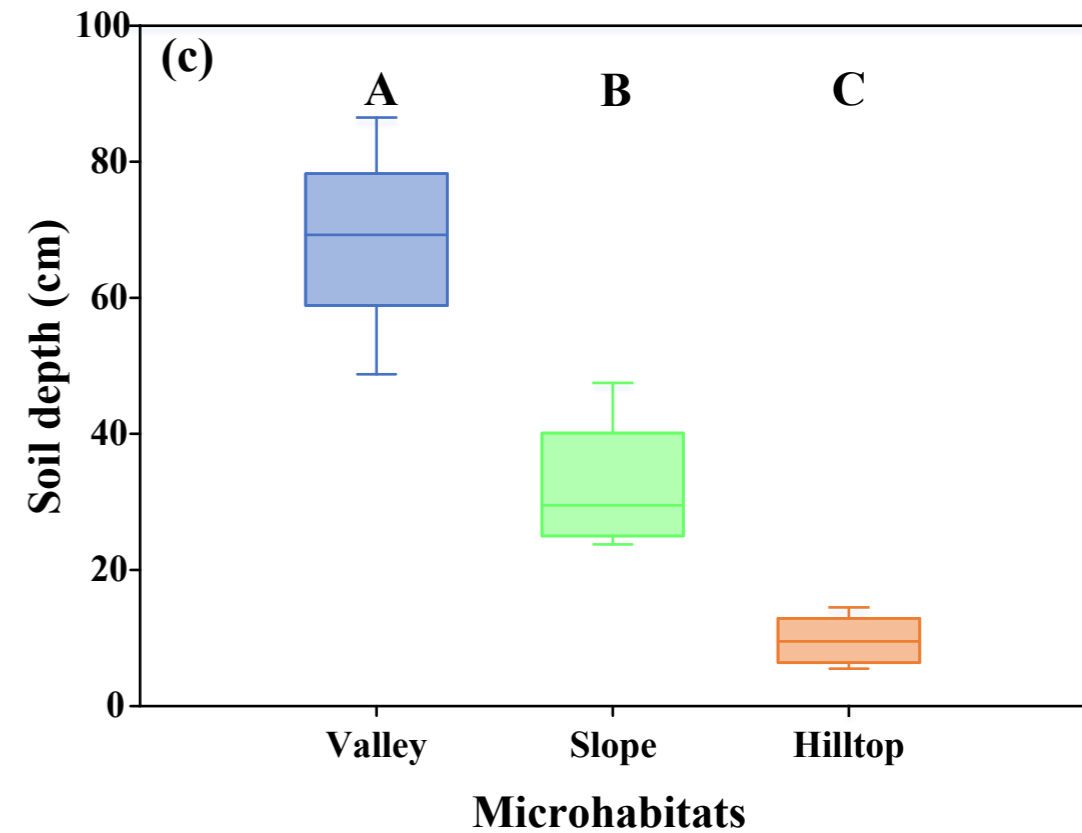

Supplement: Supplementary file 1 — Figure S1: Edaphic characteristics across the three microhabitats. (a) Volumetric soil water content measured at 5–10 cm depth during the dry season; (b) Percentage of bedrock exposure visually estimated within each microhabitat; (c) Soil depth measured from the surface to the refusal layer. Different lowercase letters above the boxes indicate significant differences among microhabitats (p < 0.05). [file ECE3-15-e72744-s005.pdf]

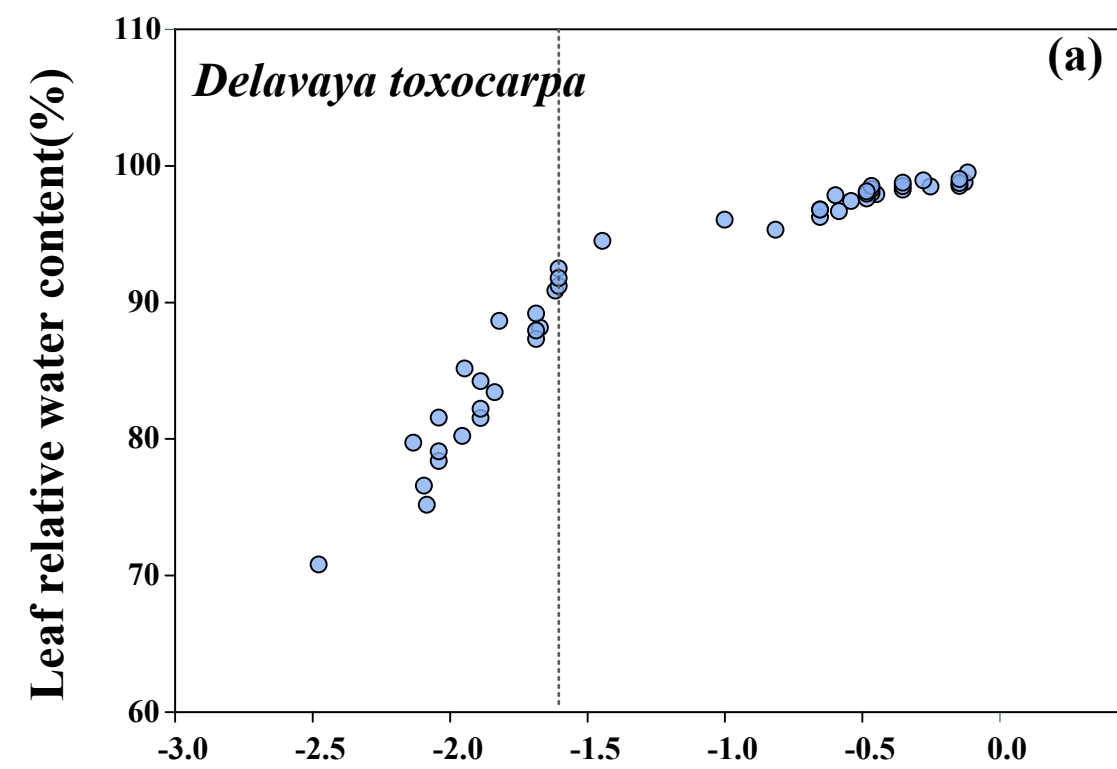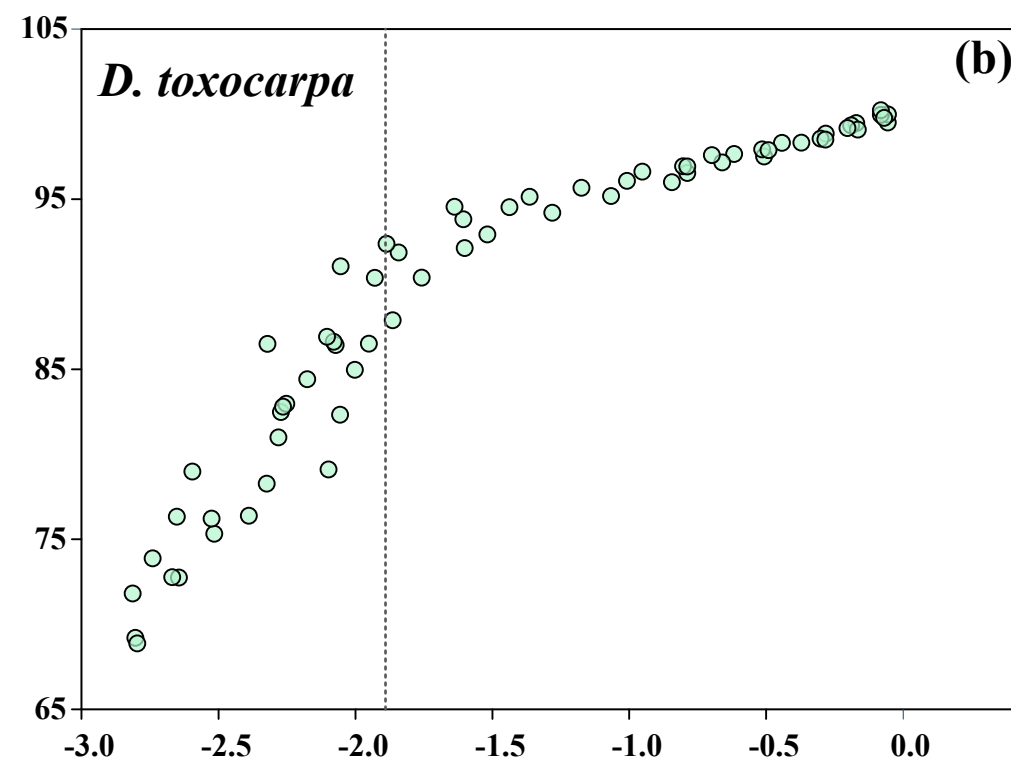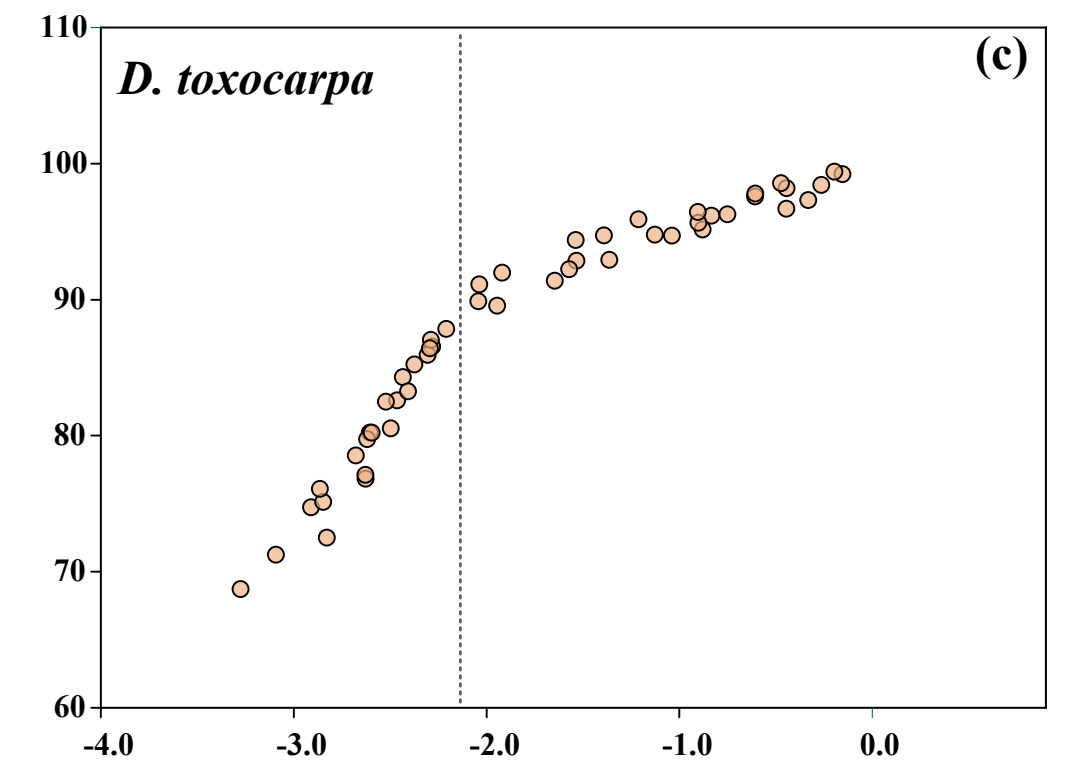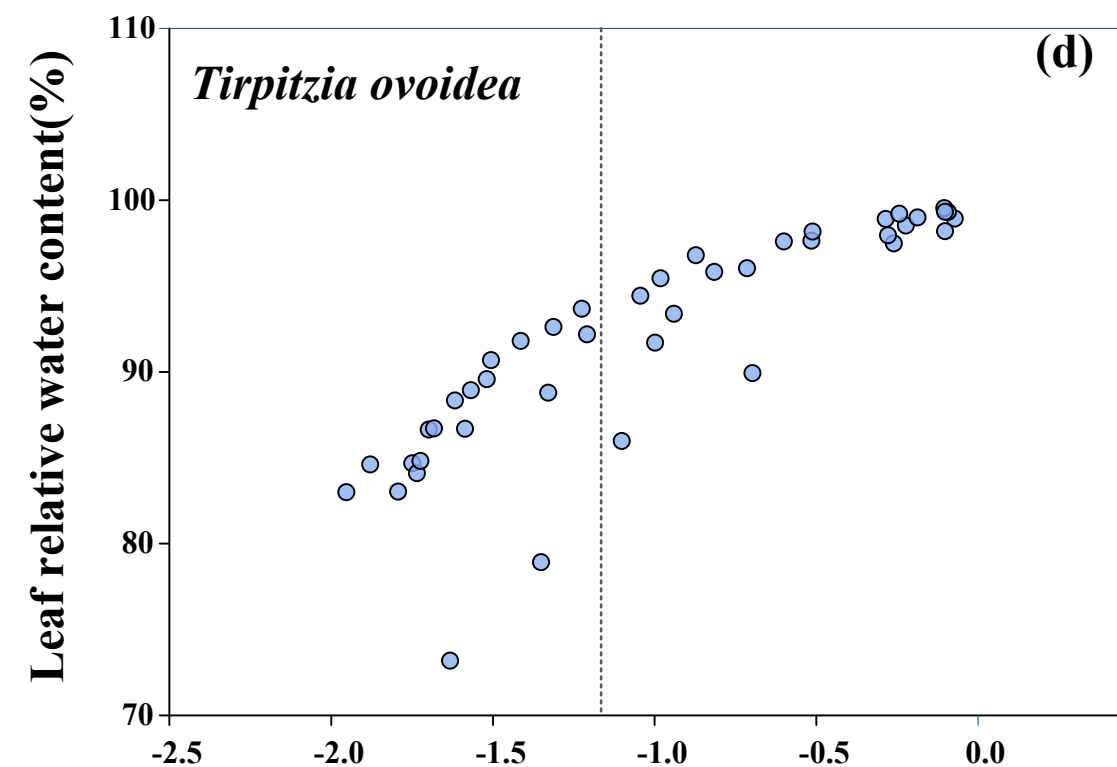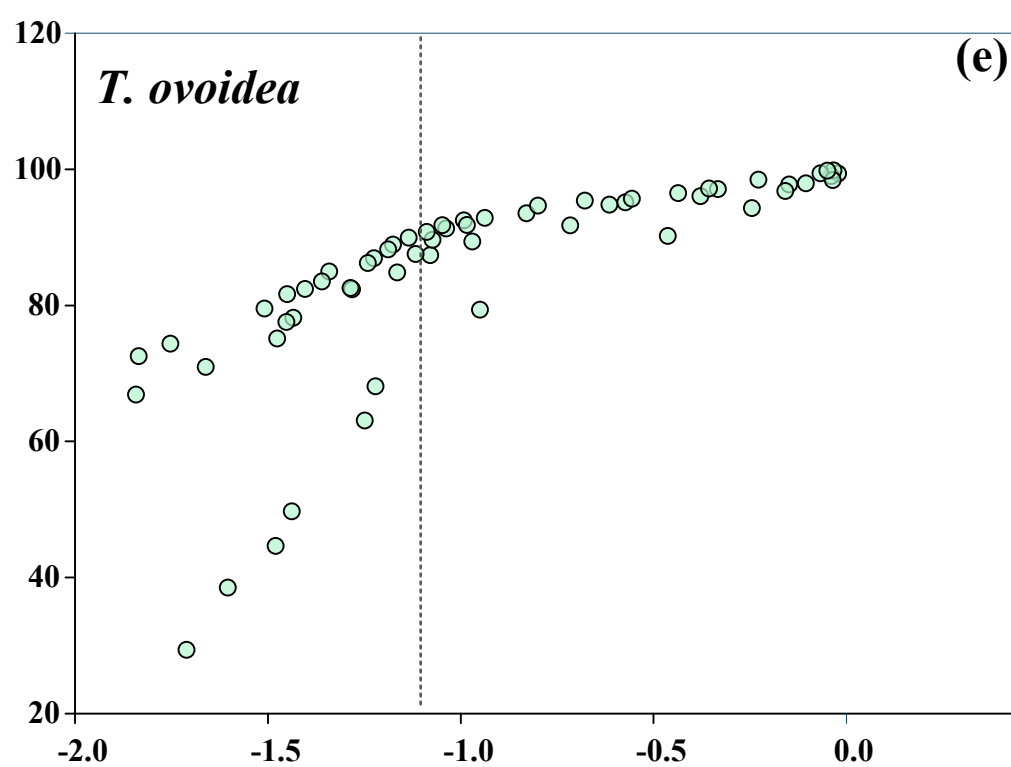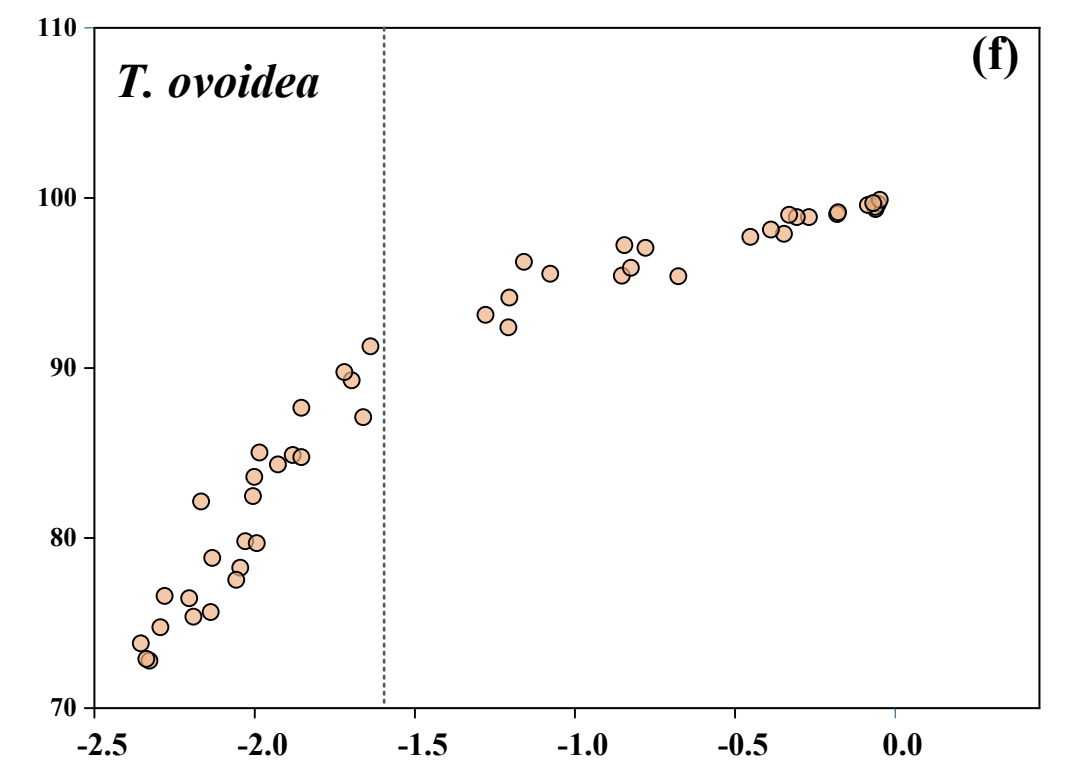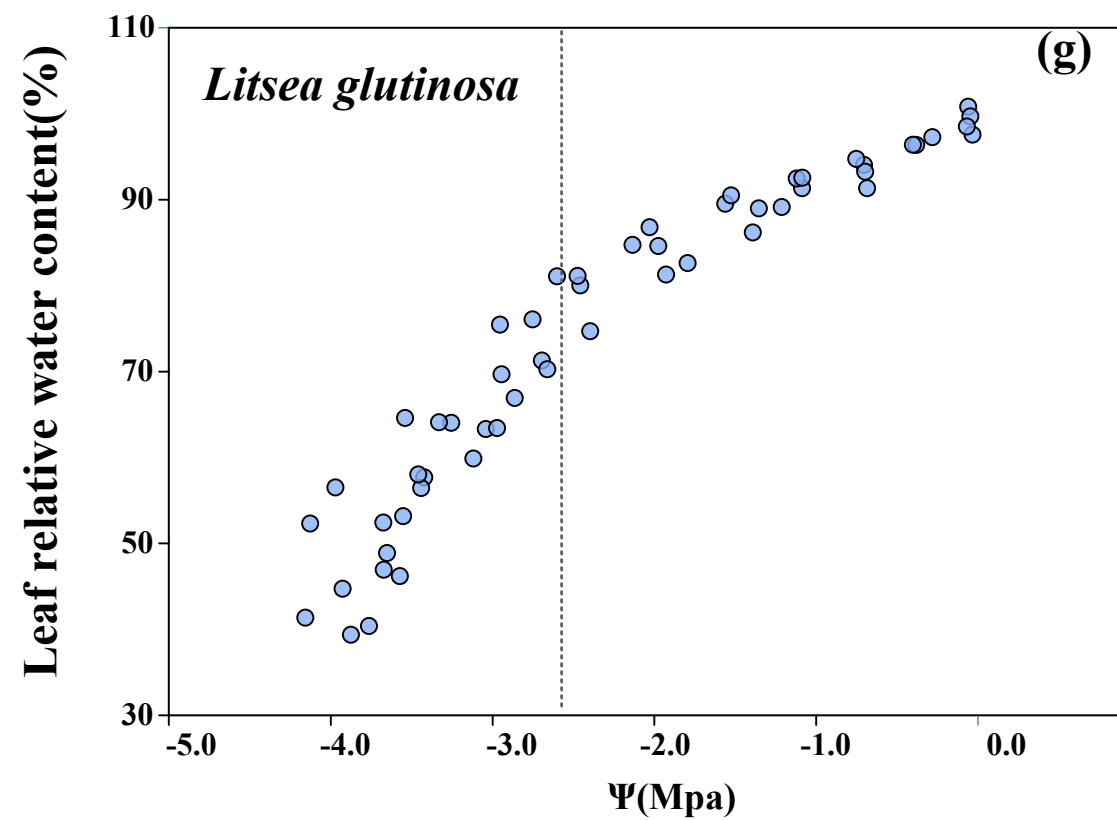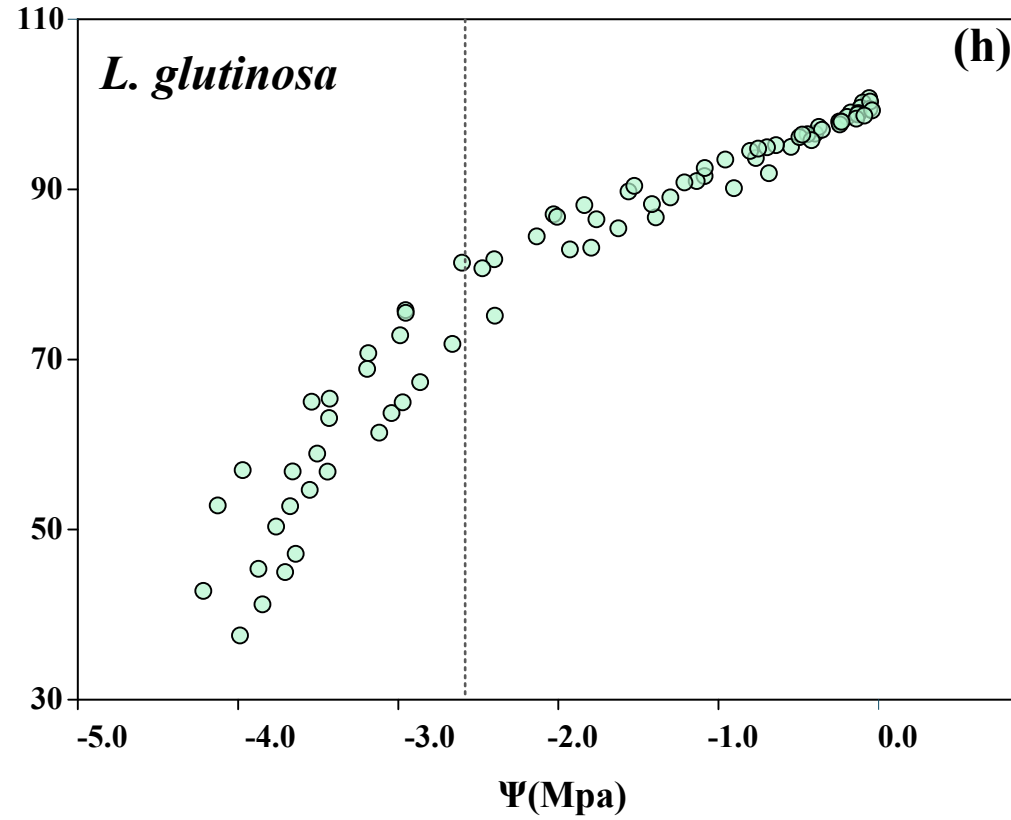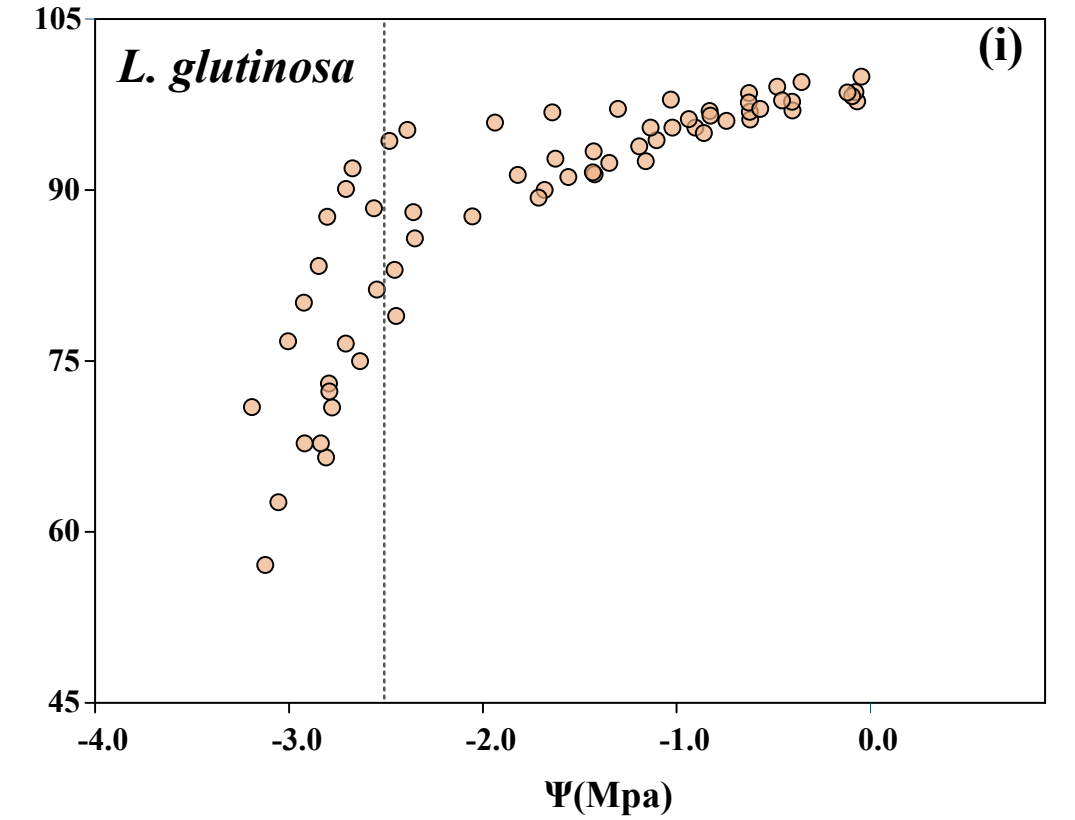

Supplement: Supplementary file 2 — Figure S2: Pressure–volume curves for (a, b, c) Delavaya toxocarpa, (d, e, f) Tirpitzia ovoidea, and (g, h, i) Litsea glutinosa. Microhabitats are distinguished by color: valley (blue), slope (green), hilltop (yellow). The dashed vertical line denotes the leaf water potential at turgor loss point. [file ECE3-15-e72744-s006.pdf]

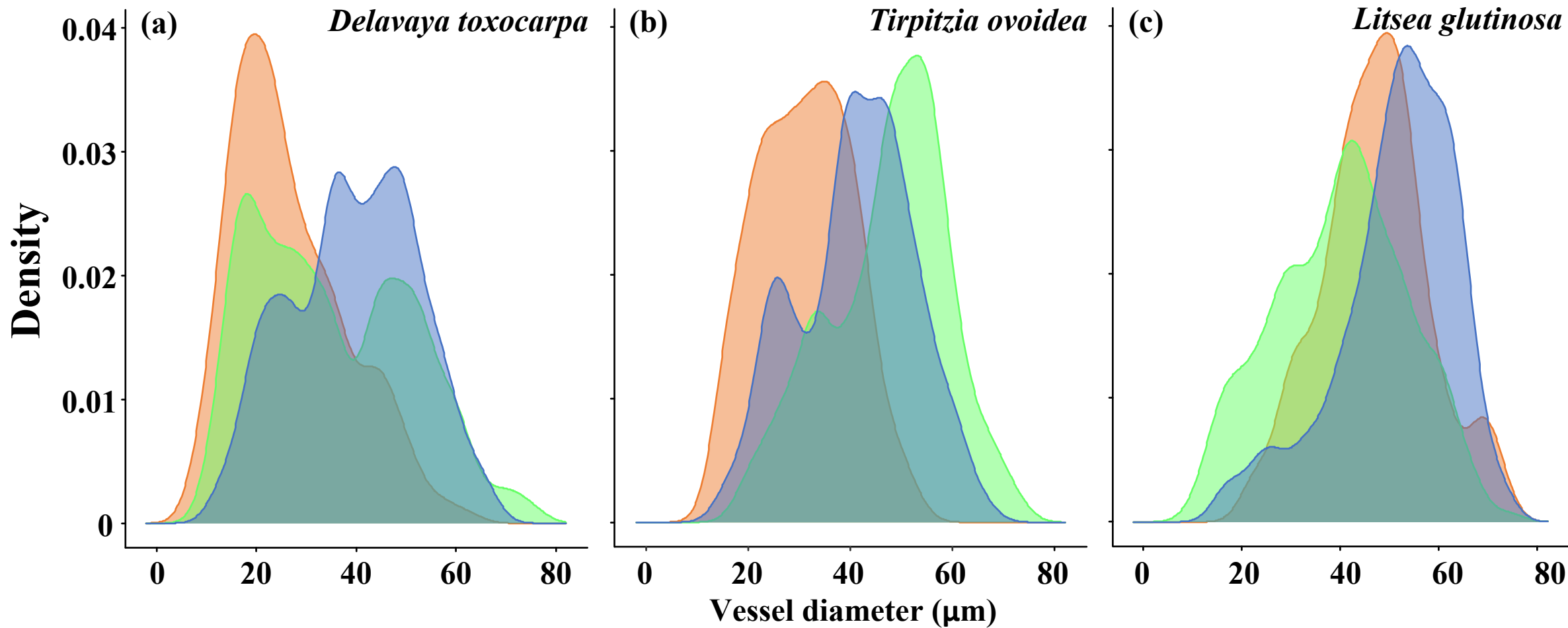

Supplement: Supplementary file 3 — Figure S3: Frequency distribution of vessel diameters of (a) D. toxocarpa; (b) T. ovoidea ; and (c) L. glutinosa . Orange, green, and blue indicate hilltop, slope, and foot, respectively. [file ECE3-15-e72744-s002.pdf]

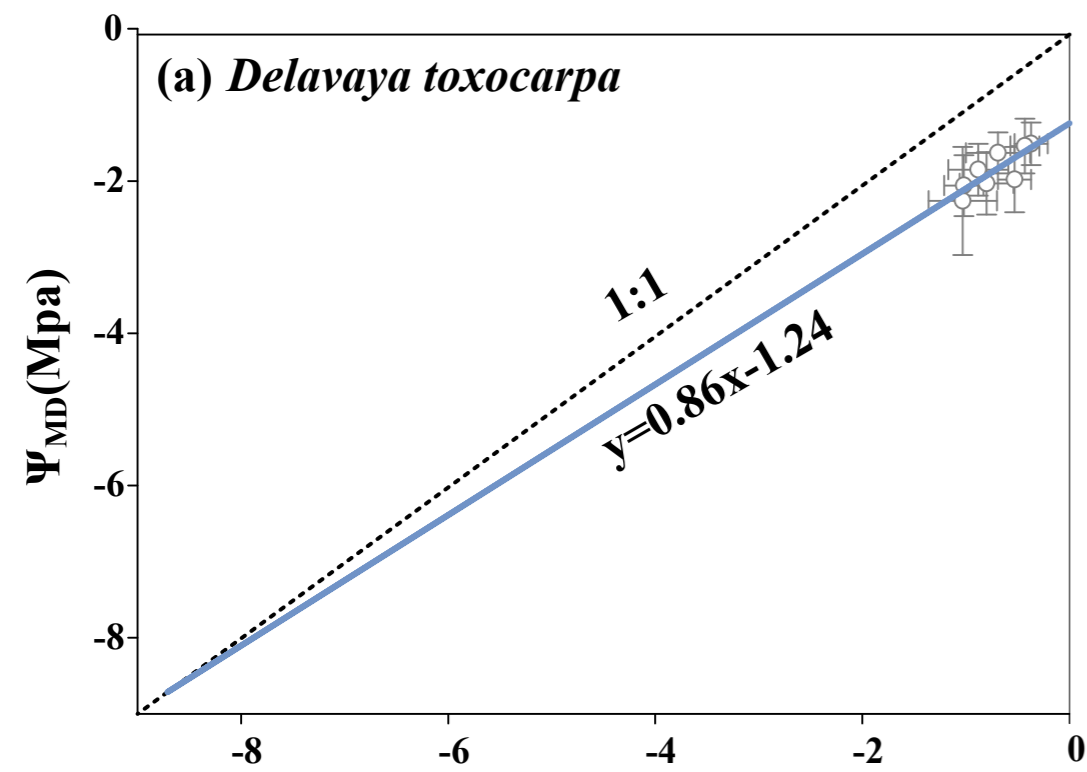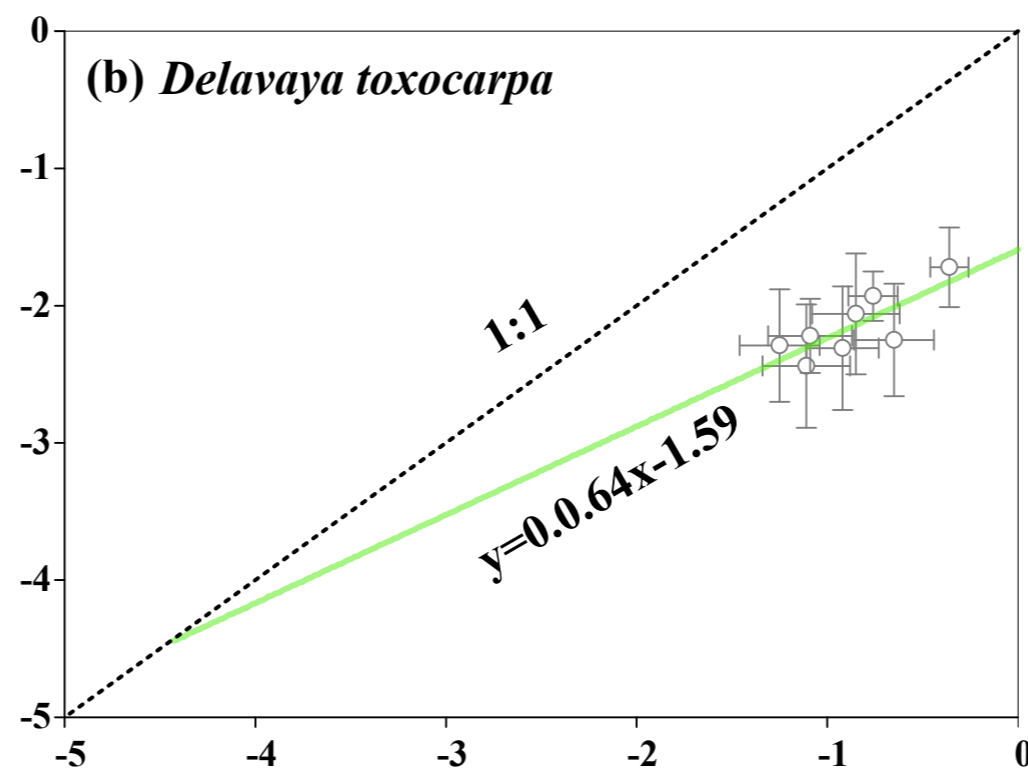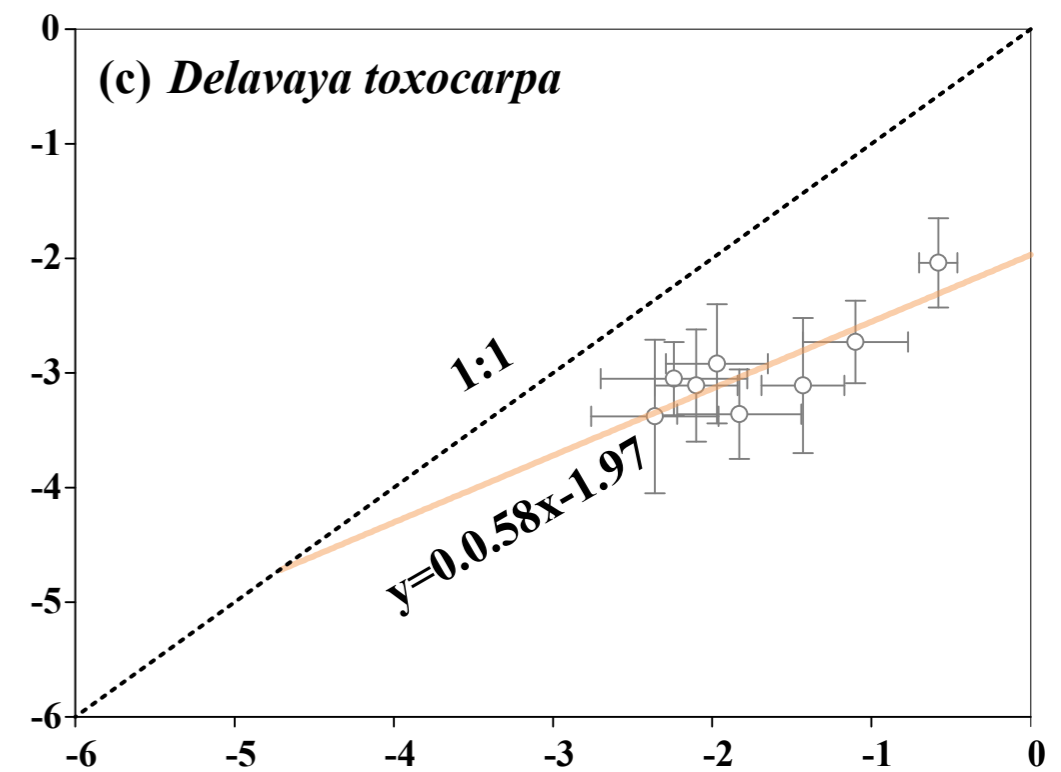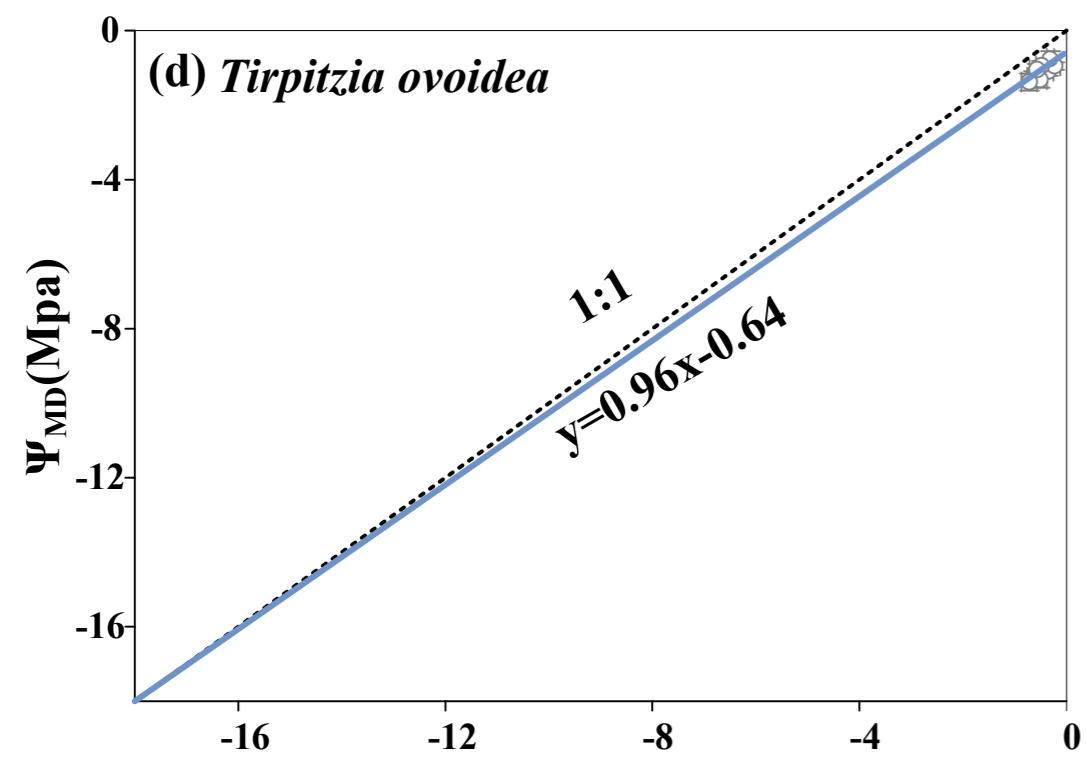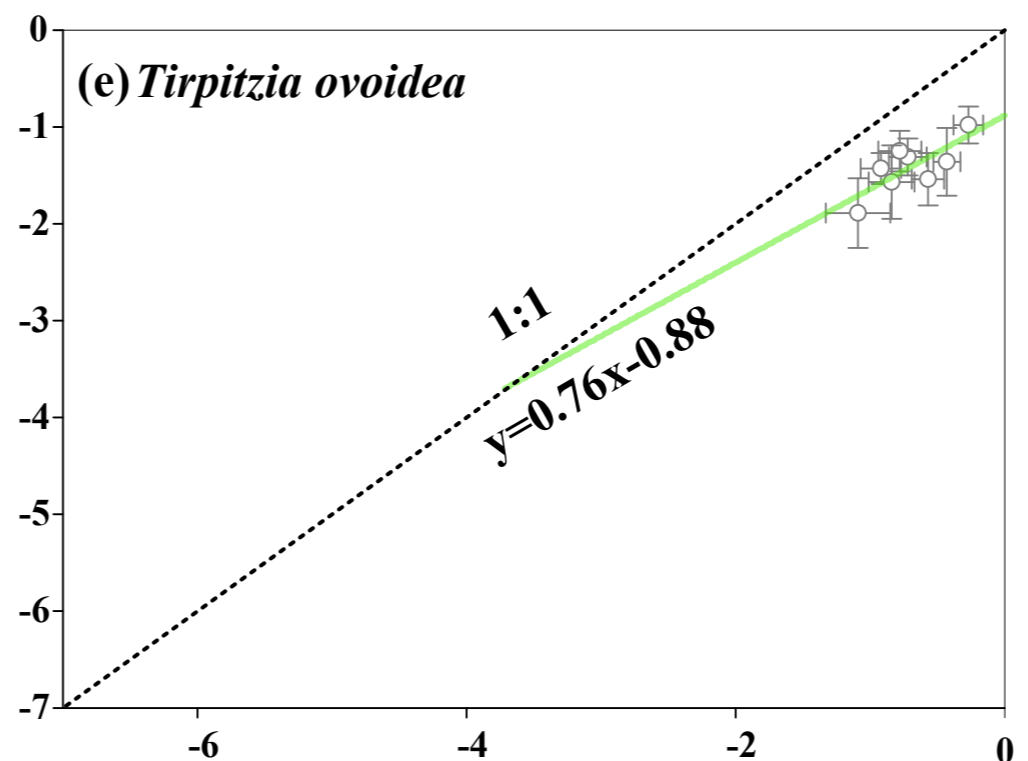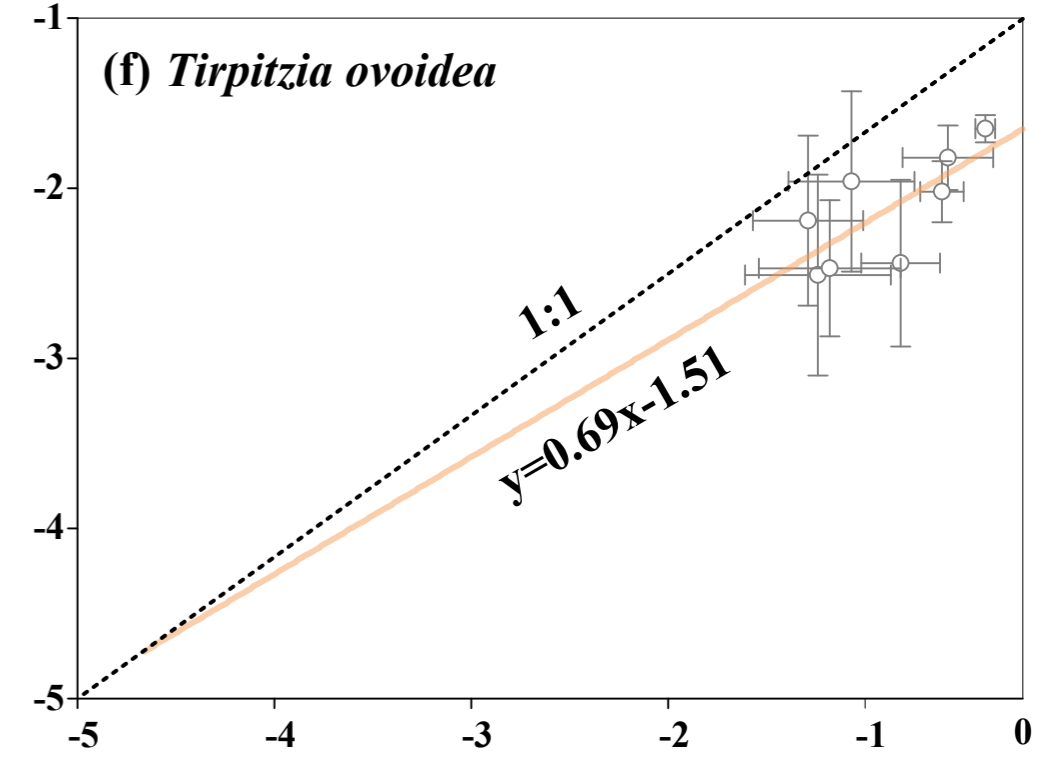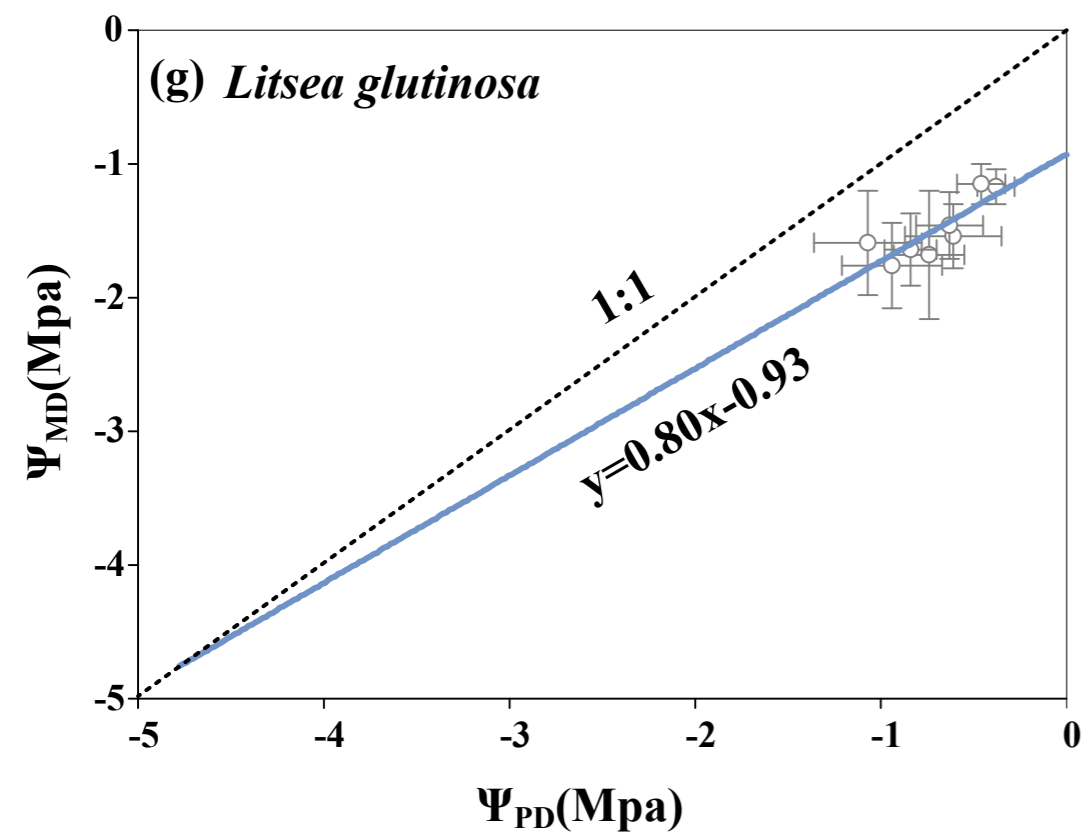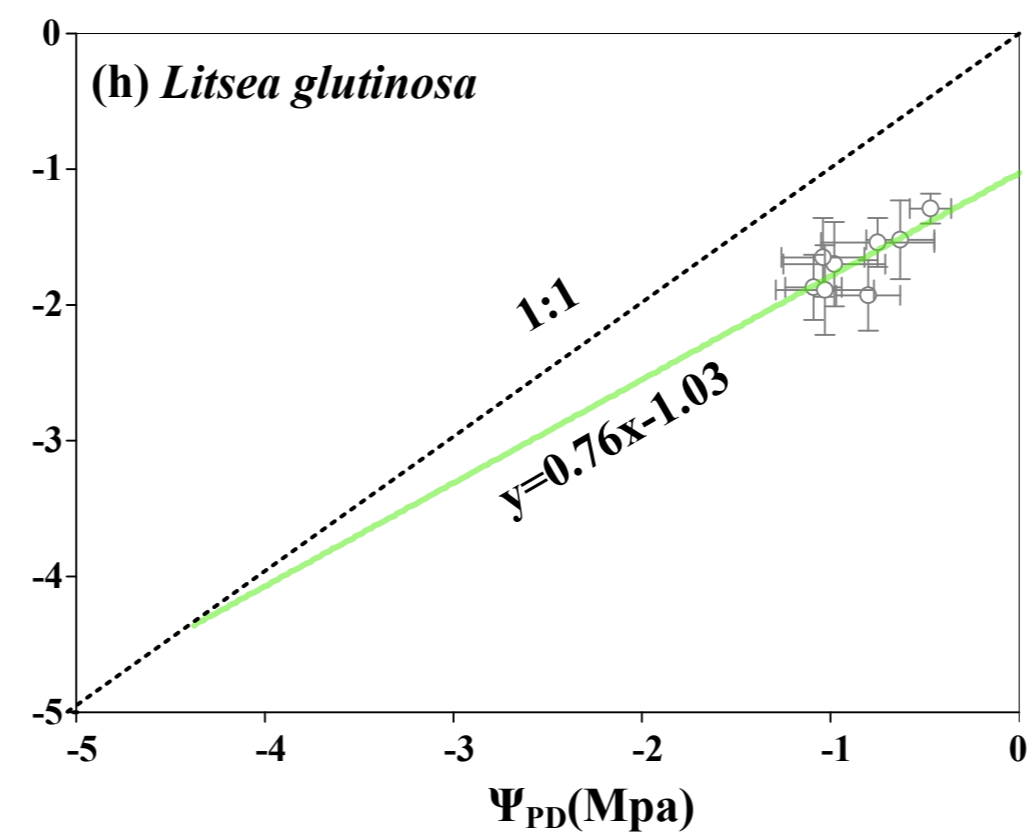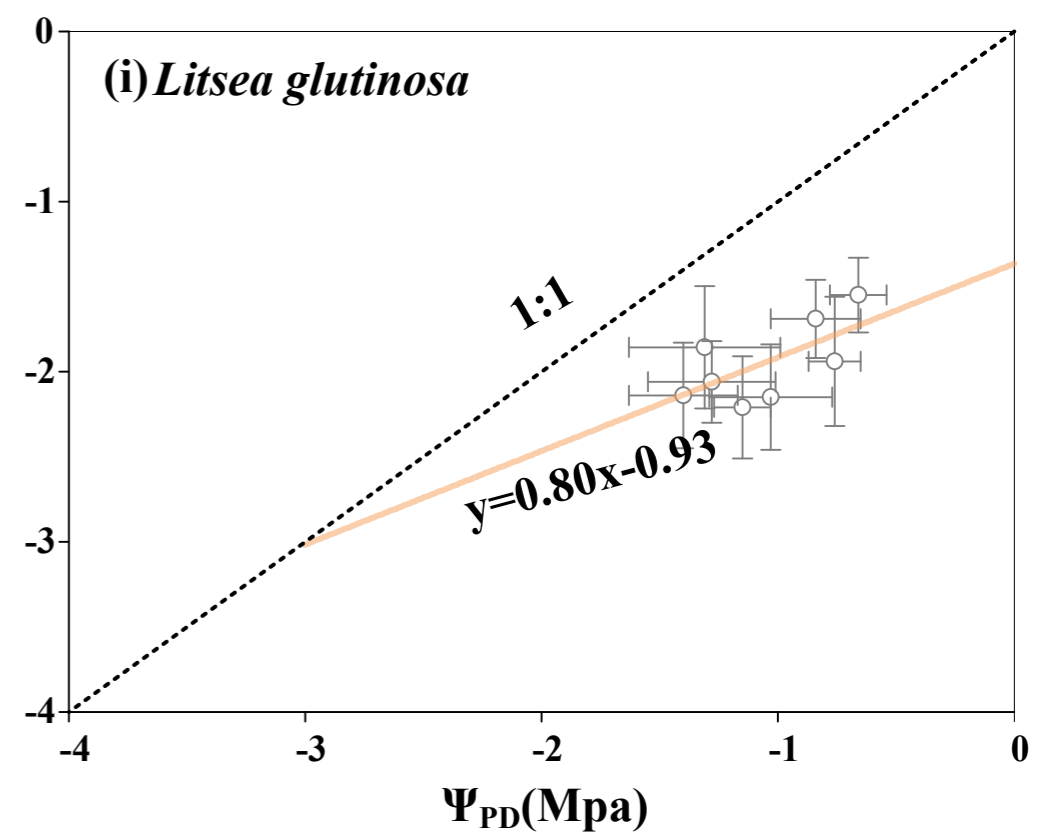

Supplement: Supplementary file 4 — Figure S4: Hydroscape area for each species across the three microhabitats. Panels (a, b, c) represent Delavaya toxocarpa, (d, e, f) Tirpitzia ovoidea, and (g, h, i) Litsea glutinosa. Microhabitats are distinguished by color: valley (blue), slope (green), hilltop (yellow). [file ECE3-15-e72744-s004.pdf]
