# Supplementary material for: Complementary Strategies of Hydraulic Variability and Conservative Stomatal Regulation Enable Widespread Distributions in a Heterogeneous Karst Landscape
Source: Ecol Evol. 2025 Dec 17;15(12):e72744. doi: 10.1002/ece3.72744 (PMC12711600; doi:10.1002/ece3.72744)
Supplement: Supplementary file 5 — Data S1: Methods. [file ECE3-15-e72744-s003.docx]

**Methods for Soil Parameter Measurement**

Soil parameters were quantified through systematic field surveys within randomly established 10 m × 10 m plots in each microhabitat type, with five plots surveyed per microhabitat. Each plot was subdivided into 5 m × 5 m subplots. Within each subplot, bedrock exposure was visually estimated, and soil depth was measured centrally using a soil auger, shifting to a nearby accessible location if the center was bedrock-exposed.

Volumetric soil water content was determined from topsoil samples collected at 5–10 cm depth during the dry season. Soil cores were extracted with a 2 cm diameter hand auger, immediately sealed in pre-weighed airtight aluminum tins, and weighed to obtain fresh mass. Samples were oven-dried at 105°C for 10 hours, then re-weighed to determine dry mass. Volumetric soil water content was calculated from the mass difference relative to the dry mass.
