# Supplementary material for: Complementary Strategies of Hydraulic Variability and Conservative Stomatal Regulation Enable Widespread Distributions in a Heterogeneous Karst Landscape
Source: Ecol Evol. 2025 Dec 17;15(12):e72744. doi: 10.1002/ece3.72744 (PMC12711600; doi:10.1002/ece3.72744)
Supplement: Supplementary file 6 — Table S1: Basic characteristics of sampled trees, sample size, and measured maximum vessel length across microhabitats. [file ECE3-15-e72744-s007.docx]

**Supplementary Table S1.** Basic characteristics of sampled trees, sample size, and measured maximum vessel length across microhabitats.

| Species | Family | Growth | Microhabitat | DBH (cm) | Height (m) | n_TOA_ | n_PLA_ | MVL |
| --- | --- | --- | --- | --- | --- | --- | --- | --- |
|  |  |  | Valley | 15.2 ± 1.6 | 4.0 ± 0.5 | 5 | 39 | 107.5 |
| *D. toxocarpa* | Sapindaceae | Shrub | Slope | 12.2 ± 2.4 | 3.7 ± 0.6 | 5 | 42 | 100 |
|  |  |  | Hilltop | 11.2 ± 1.3 | 3.3 ± 0.3 | 5 | 39 | 86.5 |
|  |  |  | Valley | 7.7 ± 1.4 | 1.8 ± 0.2 | 5 | 34 | 67 |
| *T. ovoidea* | Linaceae | Shrub | Slope | 7.8 ± 0.6 | 1.8 ± 0.2 | 5 | 35 | 56 |
|  |  |  | Hilltop | 6.3 ± 0.7 | 1.6 ± 0.2 | 5 | 51 | 41 |
|  |  |  | Valley | 7.3 ± 1.4 | 3.4 ± 0.6 | 5 | 46 | 58.5 |
| *L. glutinosa* | Lauraceae | Small tree | Slope | 6.2 ± 1.2 | 3.1 ± 0.6 | 5 | 39 | 66.5 |
|  |  |  | Hilltop | 5.6 ± 0.9 | 2.7 ± 0.5 | 5 | 40 | 56 |

Note: n_TOA_, the number of branches used for measuring TLP and stem xylem anatomical traits; n_PLC_, the number of branches used for constructing vulnerability curves.
