# Supplementary material for: Complementary Strategies of Hydraulic Variability and Conservative Stomatal Regulation Enable Widespread Distributions in a Heterogeneous Karst Landscape
Source: Ecol Evol. 2025 Dec 17;15(12):e72744. doi: 10.1002/ece3.72744 (PMC12711600; doi:10.1002/ece3.72744)
Supplement: Supplementary file 7 — Table S2: Summary of hydraulic traits and variability indices for three woody species across microhabitat gradients. [file ECE3-15-e72744-s001.docx]

Table S2 The hydraulic traits in three species among different slope positions

| species | microhabitsts | *LA* | *LMA* | Ψ_TLP_ | *D*_h_ | *K*_s_ | Ψ_12stem_ | Ψ_50stem_ | *HSM*_12_ | *HSM*_50_ | σ | Hydroscapes | *PPI*_Ks_ | *PPI*_Ψ50_ |
| --- | --- | --- | --- | --- | --- | --- | --- | --- | --- | --- | --- | --- | --- | --- |
| *D. toxocarpa* | foot | 96.98^a^ | 46.33^a^ | -1.60^a^ | 45.08^a^ | 3.60^a^ | -0.80^a^ | -1.60^a^ | -0.73 | 0.07 | 0.86 | 5.37 | 0.41 | 0.49 |
|  | slope | 74.80^b^ | 91.71^b^ | -1.89^b^ | 43.89^a^ | 3.12^ab^ | -0.79^a^ | -1.46^a^ | -0.93 | -0.26 | 0.65 | 3.55 |  |  |
|  | hilltop | 36.84^c^ | 92.60^b^ | -2.17^c^ | 33.32^b^ | 2.11^b^ | -2.02^b^ | -2.88^b^ | -0.59 | 0.27 | 0.58 | 4.66 |  |  |
| *T. ovoidea* | foot | 10.23^a^ | 92.76^a^ | -1.19^a^ | 45.60^a^ | 2.70^a^ | -0.67^a^ | -1.49^a^ | 0.02 | 0.84 | 0.95 | 4.52 | 0.30 | 0.65 |
|  | slope | 8.64^a^ | 93.56^a^ | -1.06^a^ | 43.65^a^ | 2.17^ab^ | -0.88^a^ | -1.53^a^ | -0.29 | 0.36 | 0.75 | 1.62 |  |  |
|  | hilltop | 6.70^b^ | 113.88^b^ | -1.61^b^ | 34.41^b^ | 1.90^b^ | -2.14^b^ | -4.09^b^ | 0.07 | 2.02 | 0.68 | 3.68 |  |  |
| *L. glutinosa* | foot | 41.65 | 85.58 | -2.51 | 54.23 | 2.28 | -1.27 | -2.25 | -0.07 | 0.91 | 0.80 | 2.19 | 0.14 | 0.14 |
|  | slope | 41.26 | 91.19 | -2.55 | 46.06 | 1.96 | -1.18 | -2.15 | -0.41 | 0.56 | 0.76 | 2.21 |  |  |
|  | hilltop | 39.91 | 88.62 | -2.49 | 50.74 | 2.09 | -0.88 | -2.49 | -0.88 | 0.73 | 0.55 | 2.07 |  |  |
